# Novel Anticancer NHC*-Gold(I) Complexes Inspired by Lepidiline A

**DOI:** 10.3390/molecules25153474

**Published:** 2020-07-30

**Authors:** Danielle Curran, Helge Müller-Bunz, Sofia I. Bär, Rainer Schobert, Xiangming Zhu, Matthias Tacke

**Affiliations:** 1 School of Chemistry, University College Dublin, Belfield, Dublin 4, Ireland; danielle.curran@ucdconnect.ie (D.C.); helge.muellerbunz@ucd.ie (H.M.-B.); xiangming.zhu@ucd.ie (X.Z.); 2 Organic Chemistry Laboratory, University of Bayreuth, Universitätsstr., 30-95440 Bayreuth, Germany; sofia.baer@uni-bayreuth.de (S.I.B.); rainer.schobert@uni-bayreuth.de (R.S.)

**Keywords:** gold, *N*-heterocyclic carbene, anticancer drug, aurophilic interactions, cytotoxicity, Lepidiline A

## Abstract

*N*-Heterocyclic carbene gold(I) complexes derived from 1,3-dibenzyl-4,5-diphenylimidazol-2-ylidene (NHC*) represent a promising class of anticancer drugs. Complexes of the type NHC*-Au-L (L = Br^−^, I^−^, C≡C-R) and [NHC*-Au-L]^+^ (L = NHC*, PPh_3_) have been synthesised. The X-ray crystal structures of all gold(I) complexes are presented; aurophilic interactions were observed in five of the complexes. The anticancer activity was assessed via MTT (3-(4,5-dimethylthiazol-2-yl)-2,5-diphenyltetrazolium bromide)-based proliferation assays against the human colon carcinoma cell line HCT-116^wt^ and the multidrug-resistant human breast carcinoma cell line MCF-7^topo^. Most complexes showed good cytotoxicity with IC_50_ values in the low micromolar range, while excellent sub-micromolar activity was observed for **2c**, **3a** and **3b**. Generally, the activity of the ligands studied was as follows: carbene > phosphine > alkyne > halide, with an exception for the highly active iodido derivative **2c**.

## 1. Introduction

**Figure 1:**
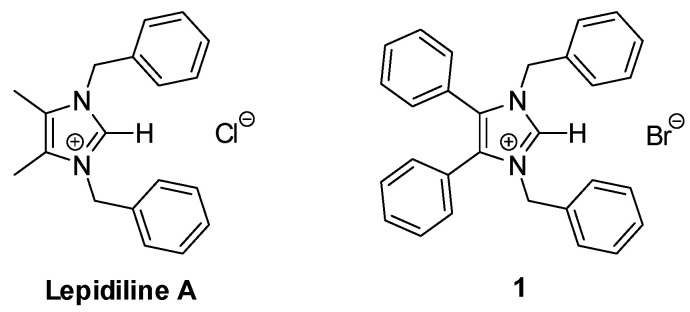
Natural product Lepidiline A and 1,3-dibenzyl-4,5-diphenylimidazolium bromide, **1**.

In recent years, *N*-heterocyclic carbenes (NHCs) have been at the forefront of anticancer drug development [1,2]. NHCs are desirable due to their high stability, versatility and lipophilicity [3]. Furthermore, they have demonstrated cytotoxic properties [4,5]. This is unsurprising as they are structurally analogous to the natural product Lepidiline A (Figure 1), a biologically active alkaloid isolated from *Lepidium meyenii*, the Peruvian maca root [6]. The structure of Lepidiline A (1,3-dibenzyl-4,5-dimethylimidazolium chloride) has been adapted to afford 1,3-dibenzyl-4,5-diphenylimidazolium bromide (Figure 1, **1**)**,** the ancillary ligand present in the series of gold(I) complexes in this work. The refinement of NHCs has resulted in many cytotoxic NHC-gold(I) complexes, thus confirming their eligibility as potential chemotherapeutic agents [7].

Alternatively, phosphine ligands have been widely used since auranofin, 2,3,4,6-tetra-*O*-acetyl-β-d-glucopyranosyl-1-thiolate-gold(I)-triethylphosphane, was recognised as an effective anti-arthritic drug. Phosphines and NHCs are regularly compared, however recent advances show NHCs as the favourable ligand due to their increased σ-donation [8]. Furthermore, the development of complexes bearing two NHCs, or biscarbenes, has drastically improved the activity of gold(I) complexes [9]. Other ligands commonly employed in gold(I) complexes are halides; they offer increased lipophilicity and lability [10,11]. Chloride is prevalent in gold(I) complexes, partly due to the widely used starting material dimethylsulfido gold(I) chloride. Interestingly, an iodido analogue of auranofin was shown to exhibit greater cytotoxicity than both auranofin and cisplatin against the human ovarian cancer cell line A2780 [12]. Alkynes are another ligand class often seen in gold(I) complexes, however only a handful of NHC-gold(I)-acetylides synthesised have been tested for their biological properties [13,14,15]. Mostly, gold(I)-acetylides are studied for their photophysical properties [16], although there are examples of phosphine-gold(I)-acetylides displaying high cytotoxicity against selected cancer cell lines [17,18].

Recently, we have reported the in vivo anticancer activity of two gold(I)-based complexes, NHC*-Au(I)-SC(S)N(CH_3_)_2_ and NHC*-Au(I)-(2,3,4,6-tetra-*O*-acetyl-α-d-glucopyranosyl-1-thiolate), against the human prostate cancer PC3 tumour model [19]. These two complexes were selected to compare the effect of the non-targeting and targeting moieties adjacent to the NHC*-gold(I) species. Both complexes exhibited excellent cytotoxicity; the dithiocarbamate derivative (non-targeting) showed better cell proliferation and tolerability. These results encouraged further investigation into diversifying the ligand trans to the NHC*-gold(I).

**Figure 2:**
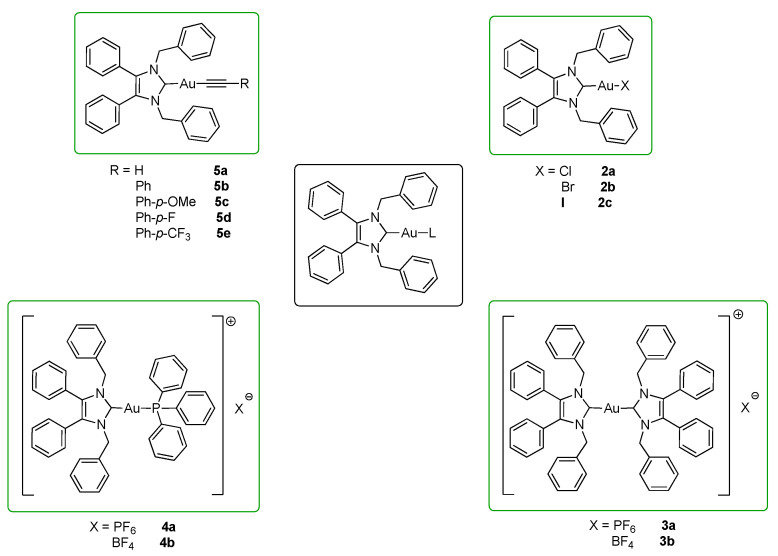
NHC*-gold(I) complexes studied in this report.

Herein, we present the synthesis, structural and biological evaluation of eleven NHC*-gold(I) complexes with a variety of ligands: halides, carbenes, phosphines and alkynes (Figure 2). X-ray crystallography was employed to discuss the bonding at the gold(I) centre and the aurophilic interactions present in the solid state. The antiproliferative properties have been determined in order to draw a structure–activity relationship for NHC*-gold(I) complexes. This series of mononuclear neutral NHC*-Au-L and cationic [NHC*-Au-L]^+^ complexes allows the effect of different ligand classes on cytotoxicity to be evaluated. Additionally, a comparison can be noted between carbenes and phosphines, and monocarbenes to biscarbenes. To the best of our knowledge, complexes **5a**–**5e** are some of the first examples of NHC-gold(I)-acetylides that have been tested for their anticancer activity.

## 2. Results and Discussion

### 2.1. Synthesis

NHC*-Au(I) complexes have been synthesised from compounds **1** and **2a**, which are previously published [20,21]. All novel gold(I) complexes were confirmed by ^1^H-NMR and ^13^C-NMR spectroscopy, ESI mass spectrometry, IR spectroscopy, X-ray crystallography and elemental analysis.

**Scheme 1:**
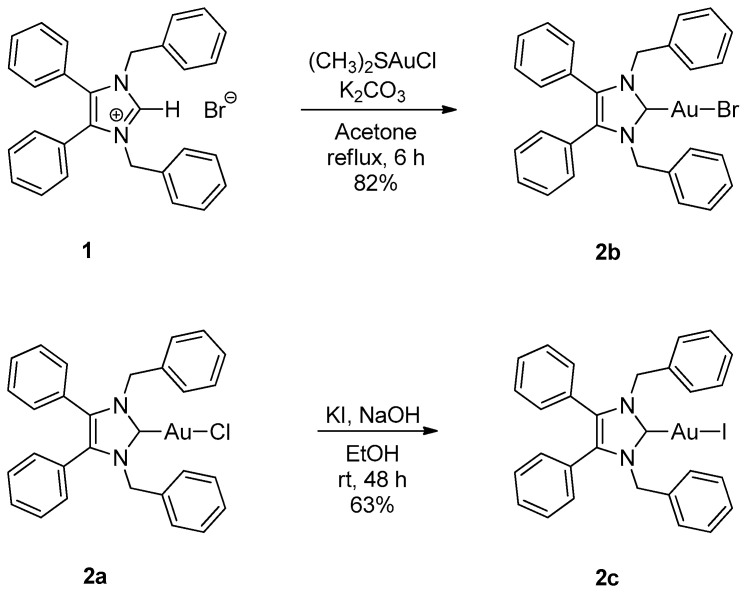
Synthesis of NHC*-Au-halides **2b** and **2c**.

NHC*-Au(I)-Br (**2b**) was synthesised via an established method [10], by reacting the imidazolium salt **1** directly with dimethylsulfido gold(I) chloride affording **2b** in a high yield (Scheme 1). Whereas the iodide analogue was formed from the already coordinated NHC*-Au(I)-Cl, **2a**, affording **2c** after a simple halide exchange with excess potassium iodide. Compound **2c** was formed in a good yield, however the product exhibited minor instability in solution when exposed to light.

The ^1^H-NMR spectra of **2b** and **2c** are identical to the chloride analogue **2a**. However, in the ^13^C-NMR spectrum the carbene carbon of **2c** appears at δ = 181.5 ppm, more downfield than both the bromide or chloride species (δ = 174.9 and 171.6 ppm, respectively). Chemical shifts are increasingly shielded as electronegativity increases, thus **2a** is the furthest upfield due to its high electronegativity and **2c** is downfield due to its lower electronegativity.

**Scheme 2:**
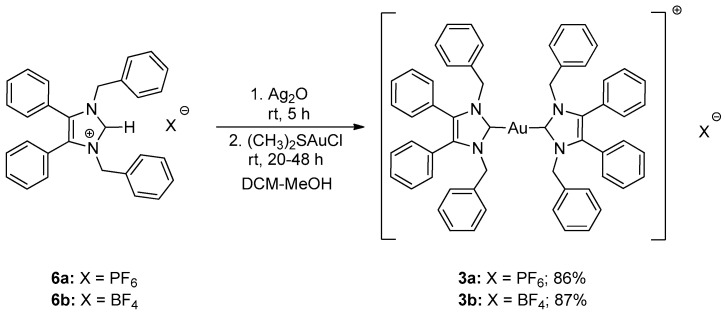
Synthesis of [NHC*_2_Au]X complexes **3a** and **3b**.

Biscarbenes **3a** and **3b** were synthesised from their corresponding imidazolium salts, **6a** and **6b**, according to literature conditions (Scheme 2) [22]. The salts were formed through anion exchange, replacing the bromide anion with hexafluorophosphate and tetrafluoroborate, in high yields of 91% and 88%. These were subsequently treated with silver oxide and later transmetallated in situ with half an equivalent of dimethylsulfido gold(I) chloride to afford biscarbenes **3a** and **3b** in high yields.

The CH_2_ moiety of the NHC ligand is a convenient spectroscopic handle to monitor the conjugation of new ligands. In their respective ^1^H-NMR spectra, the monocarbene halide complexes **2a**–**2c** all show a characteristic CH_2_ peak at δ = 5.44 ppm. The CH_2_ peaks appear at δ = 5.23 and 5.39 ppm for the imidazolium salts **6a** and **6b**, whereas upon coordination to the gold the CH_2_ peaks shift to δ = 5.19 and 5.21 ppm for biscarbenes **3a** and **3b**, respectively. Monitoring this region using ^1^H-NMR spectroscopy during the reaction enabled full conversion of these complexes to be achieved. Additionally, the disappearance of the imidazolium proton from each ^1^H-NMR spectra indicated coordination to the gold. ^19^F-NMR and ^31^P-NMR spectroscopy confirmed the presence of the anion in both the imidazolium and the conjugated biscarbene complexes.

**Scheme 3:**
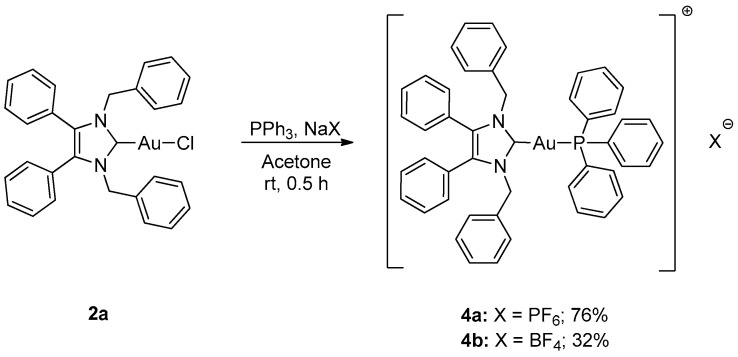
Synthesis of [NHC*-Au-PPh_3_]X complexes **4a** and **4b**.

Mixed NHC*-gold(I)-phosphine complexes **4a** and **4b** were synthesised following an established method [22]. Complex **2a** was reacted with triphenylphosphine and either NaPF_6_ or NaBF_4_ (Scheme 3). Ligand scrambling was observed with longer reaction times and degradation to [NHC*_2_Au]^+^ and PPh_3_-Au-Cl was noted. ^1^H-NMR spectroscopy confirmed the conjugation of the phosphine ligand as additional peaks were reported in the aromatic region alongside the appearance of two new peaks in the ^31^P-NMR spectra.

**Scheme 4:**
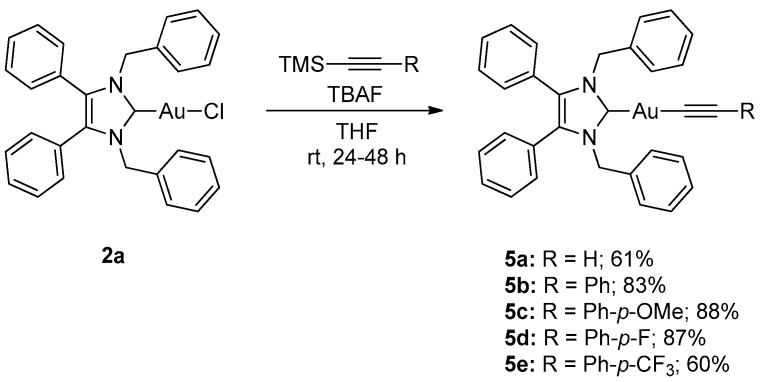
Synthesis of NHC*-Au-acetylides **5a**–**5e**.

Lastly, a series of NHC*-gold(I)-acetylides were synthesised in a procedure adapted from Mohr et al. [23]. Therein, it was demonstrated that ethynyltrimethylsilane (TMS-C≡CH) could be activated by fluoride ions present in solution by the addition of NaF. Due to solubility issues, NaF was unsuitable for the synthesis of complexes **5a**–**5e**, however, tetra-*n*-butylammonium fluoride (TBAF) in THF proved effective affording the desired complexes in good to excellent yields of 60–88% (Scheme 4). The acetylides were formed by reacting **2a** with TMS-C≡C-R in the presence of TBAF. TBAF was added dropwise as quick addition of TBAF can result in a high concentration of fluoride ions which has been reported to impede effective synthesis [24].

The IR spectra of these compounds showed the characteristic C≡C stretching at 1977 cm^−1^ for the ethynyl compound and in the 2111–2120 cm^−1^ range for the phenyl derivatives. ^1^H-NMR spectra displayed new signals for the ethynyl CH proton of **5a**, and the phenyl CH protons of **5b**–**5e**. A slight shift in the CH_2_ peak from δ = 5.44 ppm to 5.45 and 5.49 ppm, for **5a** and **5b**–**5e** respectively, confirmed conjugation. ^19^F-NMR spectroscopy proved useful as it detected the presence of the alkynyl ligand for **5d** and **5e**. More significantly, in the ^13^C-NMR spectra two signals for the alkyne appeared: δ = 121.8–125.9 ppm and 103.9–105.3 ppm. The former signal represents the carbon bonded to the gold, the latter attached to the R group on the alkyne.

### 2.2. Structural Discussion

The molecular structures of **2b**–**5e** were determined by single crystal X-ray diffraction (Figure 3 and Figure 4, Figures S27–S34), with selected bond lengths and angles displayed in Table 1. The X-ray crystal data, structure refinement and CCDC numbers are found in the supporting information (Tables S1–S4).

Compounds **2b** and **2c** crystallised in the triclinic space group P-1 (#2) with one molecule of the complex and one solvate CH_2_Cl_2_ molecule contained in the independent unit. Compounds **3a** and **5a** crystallised in the monoclinic space group P2_1_/c (#14) in the absence of solvate molecules. Compounds **3b**, **4a** and **4b** crystallised in the triclinic space group P-1 (#2) in the absence of solvate molecules. Compounds **5b** and **5e** crystallised in the monoclinic space group P2_1_/n (#14) in the absence of solvate molecules. Compound **5c** crystallised in the monoclinic space group P2_1_/n (#14) with two molecules of the compound and one solvate CH_2_Cl_2_ molecule contained in the independent unit. Compound **5d** crystallised in the monoclinic space group I2/a (#15) with four molecules of the compound and one solvate CH_2_Cl_2_ molecule contained in the independent unit. Crystals of **3a**, **3b**, **4b** and **5e** were obtained from the slow evaporation of a hot saturated ethanol solution at room temperature. Crystals of **2b**, **2c**, **4a** and **5a**–**5d** were grown by the slow infusion of pentane into a saturated CH_2_Cl_2_ solution at −18 °C.

All complexes exhibit similar Au-C bond lengths [2.000(3)–2.038(2) Å] within the expected range for these NHC-gold(I) complexes. Linear geometry at the gold(I) centre is present in all complexes, with the C(8)-Au-X bond angle in the range of 173.24(18)–179.65(16)°. First considering the halide derivatives **2b** and **2c**, the Au-X bond lengths increase as the halide descends the periodic table due to the trans effect. The Au-I bond is the longest, which directly reflects the strong trans effect of the carbene onto the halide bond. Biscarbenes **3a** and **3b** have almost identical Au-C bond lengths to both carbenes. Compounds **4a** and **4b** display Au-P bond lengths similar to those seen previously in other phosphine-gold(I) complexes [25,26].

As seen in other NHC-Au-acetylides, the bond between the gold atom and the alkynyl ligand of **5a**–**5e** is slightly shorter than that to the carbene, however still within the margins of acceptable Au-C bond lengths [14]. The C-C bond lengths of the alkyne (C_30_-C_31_) in the 1.193(6)–1.217(7) Å range are characteristic of a triple bond [27]. Furthermore, the slightly shorter C_31_-C_32_ single bond concurs with other reported gold(I)-alkynyl complexes [16].

**Figure 5:**
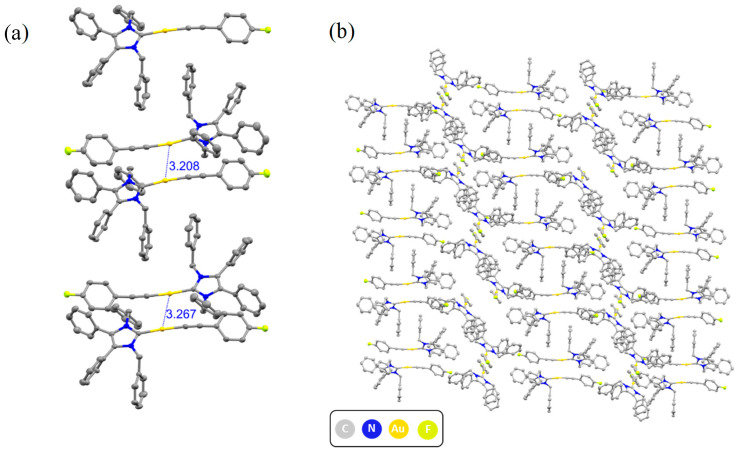
(**a**) A view along the chain of NHC*-Au-CC-Ph-F (**5d**) molecules showing the distances (marked in blue) between the gold atoms (3.2077(9) and 3.2666(9) Å. (**b**) The crystal packing of **5d** shows a pattern of parallel chains. Hydrogen atoms and solvent molecules are omitted for clarity.

Complexes **2b**, **2c**, **5a**, **5d** and **5e** exhibit aurophilic interactions in the solid state, understandably as these complexes have the smallest ligands in this report. The acetylides **5a**, **5d** and **5e** have a compact linear structure at the gold(I) centre, which causes no steric hindrance and allows for a more packed solid state structure. Unsurprisingly, the cationic complexes do not display any aurophilic bonding due to their accompanying anions being interpolated between the molecules. Compound **5d** presents the shortest Au···Au contact (Figure 5). The solid state structure of **5d** shows chains of molecules which alternate with Au···Au distances of 3.2077(9) and 3.2666(9) Å. Compound **5e** displays Au···Au interactions at a distance of 3.3318(5) Å, which is indicative of weak aurophilicity. Complexes **2b**, **2c** and **5a** report distances of 3.5242(3)–3.5850(3) Å which is at the end of the acceptable range for aurophilic character, implying very weak interactions are present in these complexes [28,29].

### 2.3. Biological Evaluation

The in vitro anticancer activity of compounds **2a**–**5e** were tested via MTT-based proliferation assays against the human colon carcinoma cell line HCT-116^wt^ and the multidrug-resistant human breast carcinoma cell line MCF-7^topo^ and compared to cisplatin and auranofin [30]. All complexes tested displayed good cytotoxicity with low micromolar activity, with the exception of some of the acetylide complexes (Table 2). Interestingly, all active complexes apart from **2a** and **2b** were more potent against the colon cancer cell line HCT-116^wt^ than the breast cancer cell line MCF-7^topo^. Complexes **2c**–**4b** were found to be more active than cisplatin and auranofin on both cell lines. Despite the lower activities of the acetylides, complex **5a** showed higher activity than cisplatin on both cell lines and a lower IC_50_ value than auranofin on the breast cancer cell line MCF-7.

Considering the series of halides, **2b** exhibited similar activity to the chloride analogue **2a**, while **2c** is significantly more active than both compounds. Compound **2c** reached sub-micromolar activity with an IC_50_ value of 0.64 ± 0.01 μM against the colon cancer cell line. Biscarbenes **3a** and **3b** exhibited similar IC_50_ values, both having sub-micromolar activity on both cell lines. The choice of the counterion did not impact the activity of complexes **3a**–**4b**. Comparing the biscarbene complexes **3a** and **3b** to the phosphine derivatives **4a** and **4b**, it is evident that the presence of a second NHC ligand contributes to a more active complex than the addition of a phosphine ligand. This supports the argument that NHCs are a superior ligand than phosphines.

Moreover, biscarbenes **3a** and **3b** can be compared to the monocarbene complexes **2a**–**2c** and **5a**–**5e**. These NHC-Au-X type complexes will dissociate under physiological conditions into [NHC-Au]^+^. Hence, it is useful to compare these monocarbenes and biscarbenes to assess the importance of a second carbene ligand. It was noted that although the NHC*-Au-I complex was very active, the monocarbenes in general had significantly lower cytotoxicity than the biscarbenes.

Finally, in relation to the acetylide complexes, **5a** and **5c** showed single-digit micromolar IC_50_ values against the colon carcinoma cell line HCT-116^wt^. On the same cell line, **5b** exhibited moderate activity, similar to **2b**. Disappointingly, compounds **5b**–**5e** reported very little activity with values above 50 μM on the breast cancer cell line; activity above 50 μM was also observed on the colon cancer cell line for **5d** and **5e**. These low activities correlate with the solubility of these acetylides and complexes **5b**, **5d** and **5e** displayed especially poor solubility. When evaluating the phenyl-based derivatives it is clear that the electronic effects of the substituents impact their cytotoxicity. Considering the HCT-116^wt^ results, this is evident as **5c** exhibited the highest activity due to its electron-donating properties and complexes **5d** and **5e** exhibited the lowest activity due to their deactivating substituents. Additionally, these IC_50_ values indicated that the addition of a phenyl group decreases the activity of these compounds as the small acetylide **5a** displayed the highest activity in the set.

In summary, good cytotoxicity was found against both the colon carcinoma cell line HCT-116^wt^ and the breast carcinoma cell line MCF-7^topo^. When compared to cisplatin and auranofin, higher activity was observed for complexes **2c**–**4b** against both cell lines. Gratifyingly, the iodide complex **2c** and the biscarbene complexes **3a** and **3b** achieved sub-micromolar activity. Overall, **3b** had the highest anticancer activity with an IC_50_ value of 0.29 ± 0.01 μM on the colon cancer cell line HCT-116^wt^. These three complexes show high promise as potential chemotherapeutics.

## 3. Materials and Methods

### 3.1. General Conditions

All chemicals were purchased and used as received, unless otherwise stated. Solvents were dried according to the standard procedures, when necessary. ^1^H, ^13^C, ^19^F and ^31^P spectra were recorded on either a 300, 400 or 500 MHz Varian spectrometer at room temperature (rt). Both chloroform (CDCl_3_) and dimethyl sulfoxide (DMSO) were used as deuterated solvents. The residual solvent peak or tetramethylsilane (TMS) were used as the internal standard. All chemical shifts are reported as δ values in parts per million (ppm). Infrared spectra were recorded on a Bruker ALPHA PLATINUM ATR spectrometer (Millerica, MA, USA). High resolution accurate mass data were obtained on either a Waters/Micromass LCT TOF spectrometer (Milford, MA, USA) or Agilent 6546 LC/Q-TOF spectrometer (Santa Clara, CA, USA) under electrospray ionisation technique. Elemental analysis was conducted on an Exeter Analytical CE-440 elemental analyser (Coventry, UK). X-Ray crystallography data were collected on a Rigaku Oxford Diffraction (Chalgrove, Oxfordshire, UK) SuperNova A diffractometer. Absorbance measurements were done with a TECAN (Mannedorf, Zurich, Switzerland) Infinite F200 plate reader.

### 3.2. Synthesis

#### 3.2.1. (1,3-Dibenzyl-4,5-diphenylimidazol-2-ylidene)gold(I) Bromide (**2b**)


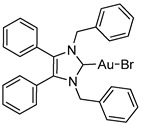


Using literature conditions [10], imidazolium bromide **1** (50 mg, 0.104 mmol), (CH_3_)_2_SAuCl (31 mg, 0.104 mmol) and K_2_CO_3_ (14 mg, 0.104 mmol) were stirred in acetone (5 mL) under reflux for 6 h. Reaction was monitored by TLC (CH_2_Cl_2_–MeOH; 7:1). The solvent was removed under reduced pressure and the crude dissolved in CH_2_Cl_2_ (15 mL). This was filtered through Celite and the solvent reduced to approximately 2 mL and pentane (40 mL) added to precipitate a white solid. This was filtered, washed with pentane (15 mL) and dried in vacuo to give a white solid (58 mg, 82%). ^1^H-NMR (400 MHz, CDCl_3_, δ ppm): 7.30 (t, *J* = 7.4 Hz, 3H, ArCH), 7.21 (m, 9H, ArCH), 7.02 (dd, *J* = 7.1, 2.1 Hz, 7H, ArCH), 6.96 (d, 4H, ArCH), 5.44 (s, 4H, CH_2_). ^13^C-NMR (101 MHz, CDCl_3_, δ ppm): 174.9 (NCN), 135.8 (C), 132.2 (C), 130.9 (CH), 129.5 (CH), 128.8 (CH), 128.7 (CH), 128.2 (CH), 127.7 (C), 127.4 (CH), 53.1 (CH_2_). HRMS (ESI^+^) *m*/*z*: [M + H]^+^ calcd. 677.0867; found 677.0847. IR (ATR, cm^−1^): 3056 (w), 3028 (w), 1604 (w), 1487 (m), 1446 (s), 1025 (m). Anal. calcd. for C_29_H_24_N_2_AuBr (677.38) in %: C, 51.42; H, 3.57; N, 4.14; Br, 11.80. Found: C, 51.59; H, 3.51; N, 4.05; Br, 11.85.

#### 3.2.2. (1,3-Dibenzyl-4,5-diphenylimidazol-2-ylidene)gold(I) Iodide (**2c**)


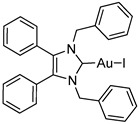


NHC*-Au(I)-Cl (**2a**) (50 mg, 0.079 mmol, 1.0 equiv.) and NaOH (4 mg, 0.079 mmol, 1.0 equiv.) were stirred with an excess of KI (65 mg, 0.395 mmol, 5.0 equiv.) in EtOH (15 mL) at rt for 48 h. The solvent was removed under reduced pressure, the crude was dissolved in CH_2_Cl_2_ (15 mL) and dried over anhydrous MgSO_4_. This was filtered and reduced to approximately 2 mL under reduced pressure. Pentane (40 mL) was added to precipitate a solid. The product was filtered, washed with pentane (15 mL) and dried in vacuo to give an off-white solid (36 mg, 63%). ^1^H-NMR (500 MHz, CDCl_3_, δ ppm): 7.30 (t, *J* =7.5 Hz, 2H, ArCH), 7.22 (m, 9H, ArCH), 7.02 (d, 4H, ArCH), 6.97 (d, 4H, ArCH), 5.44 (s, 4H, CH_2_). ^13^C-NMR (101 MHz, CDCl_3_, δ ppm): 181.5 (NCN), 135.6 (C), 131.9 (C), 130.8 (CH), 130.7 (CH), 129.4 (CH), 128.6 (CH), 128.1 (CH), 127.6 (C), 127.3 (CH), 52.7 (CH_2_). HRMS (ESI^+^) *m*/*z*: [M + Na]^+^ calcd. 747.0548; found 747.0541. IR (ATR, cm^−1^): 3062 (w), 3028 (w), 1603 (w), 1494 (m), 1447 (s), 1023 (m). Anal. calcd. for C_29_H_24_N_2_AuI (724.38) in %: C, 48.09; H, 3.34; N, 3.87. Found: C, 47.80; H, 3.18; N, 3.74.

#### 3.2.3. 1,3-Dibenzyl-4,5-diphenylimidazolium Hexafluorophosphate (**6a**)


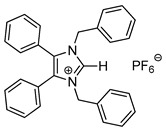


Using literature conditions [22], imidazolium bromide **1** (250 mg, 0.520 mmol, 1.0 equiv.) and NaPF_6_ (135 mg, 0.800 mmol, 1.5 equiv.) were stirred in acetone (30 mL) at rt for 24 h. The solvent was removed under reduced pressure and the crude dissolved in CH_2_Cl_2_ (15 mL). This was filtered and the solvent reduced to approximately 2 mL and pentane (40 mL) added to precipitate a white solid. This was filtered, washed with pentane (15 mL) and dried in vacuo to give a white solid (258 mg, 91%). ^1^H-NMR (400 MHz, CDCl_3_, δ ppm): 8.56 (s, 1H, CH_imidazole_), 7.35 (m, 11H, ArCH), 7.21 (d, *J* = 7.0 Hz, 4H, ArCH), 7.06 (m, 4H, ArCH), 5.23 (s, 4H, CH_2_). ^19^F-NMR (376 MHz, CDCl_3_, δ ppm): −71.76, −73.65. ^31^P-NMR (162 MHz, CDCl_3_, δ ppm): 62.90, −135.44, −139.84, −144.25, −148.65, −153.06. ^13^C-NMR (101 MHz, CDCl_3_, δ ppm): 135.3 (C), 133.0 (CH), 132.7 (C), 130.9 (CH), 130.6 (CH), 129.4 (CH), 129.3 (CH), 129.2 (CH), 128.6 (CH), 124.7 (C), 51.9 (CH_2_). HRMS (ESI^+^) *m*/*z*: [M − PF_6_]^+^ calcd. 401.2012; found 401.2012. IR (ATR, cm^−1^): 3077, 2923, 1603, 1497, 1451, 1022. Anal. calcd. for C_29_H_25_N_2_PF_6_ (546.49) in %: C, 63.74; H, 4.61; N, 5.13; F, 20.86. Found: C, 63.72; H, 4.59; N, 5.09; F, 20.52.

#### 3.2.4. 1,3-Dibenzyl-4,5-diphenylimidazolium Tetrafluoroborate (**6b**)


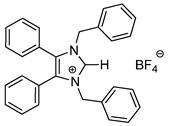


In a procedure identical to above reaction: Imidazolium bromide **1** (250 mg, 0.520 mmol), NaBF_4_ (88 mg, 0.800 mmol, 1.5 equiv.) and acetone (30 mL). After workup and precipitation a white solid was formed (224 mg, 88%). ^1^H-NMR (400 MHz, CDCl_3_, δ ppm): 9.94 (s, 1H, CH_imidazole_), 7.40 (m, 2H, ArCH), 7.32 (m, 6H, ArCH), 7.25 (m, 5H, ArCH), 7.14 (dd, *J* = 8.6, 1.7 Hz, 4H, ArCH), 7.07 (m, 6H, ArCH), 5.39 (s, 4H, CH_2_). ^19^F-NMR (376 MHz, CDCl_3_, δ ppm): −151.89, −151.95. ^13^C-NMR (101 MHz, CDCl_3_, δ ppm): 136.8 (C), 133.2 (CH), 132.6 (C), 130.9 (CH), 130.5 (CH), 129.3 (CH), 129.2 (CH), 129.1 (CH), 128.6 (CH), 124.9 (C), 51.7 (CH_2_). HRMS (ESI^+^) *m*/*z*: [M − BF_4_]^+^ calcd. 401.2012; found 401.2012. IR (ATR, cm^−1^): 3069, 2913, 1605, 1497, 1452, 1020. Anal. calcd. for C_29_H_25_N_2_BF_4_ (488.34) in %: C, 71.33; H, 5.16; N, 5.74; F, 15.56. Found: C, 71.22; H, 5.11; N, 5.97; F, 10.27.

#### 3.2.5. Bis-[1,3-dibenzyl-4,5-diphenylimidazol-2-ylidene]gold(I) Hexafluorophosphate (**3a**)


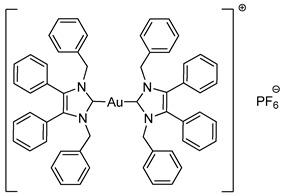


Using literature conditions [22], imidazolium salt **6a** (50 mg, 0.092 mmol, 1.0 equiv.) and Ag_2_O (23 mg, 0.101 mmol, 1.1 equiv.) were stirred in CH_2_Cl_2_-MeOH (1:1, 30 mL) in darkness for 5 h. (CH_3_)_2_SAuCl (13 mg, 0.046 mmol, 0.5 equiv.) was added and the reaction stirred for a further 20 h. The solvent was removed under reduced pressure. The residue was dissolved in CH_2_Cl_2_ (15 mL) and filtered through Celite. The solvent was reduced to approximately 2 mL and pentane (40 mL) was added to precipitate the product. This was filtered, washed with pentane (15 mL) and dried in vacuo to give a white solid (45 mg, 86%). ^1^H-NMR (400 MHz, CDCl_3_, δ ppm): 7.28 (d, 2H, ArCH), 7.17 (m, 20H, ArCH), 6.99 (m, 8H, ArCH), 6.88 (dd, *J* = 7.0, 2.1 Hz, 8H, ArCH), 5.19 (s, 8H, CH_2_). ^13^C-NMR (101 MHz, CDCl_3_, δ ppm): 184.8 (NCN), 136.6 (C), 132.9 (C), 130.8 (CH), 129.5 (CH), 128.8 (CH), 127.9 (CH), 127.1 (CH), 126.8 (CH), 52.4 (CH_2_). ^19^F-NMR (376 MHz, CDCl_3_, δ ppm): −72.31, −74.20. ^31^P-NMR (162 MHz, CDCl_3_, δ ppm): 62.90, −135.39, −139.79, −144.20, −148.61, −153.01. HRMS (ESI^+^) *m*/*z*: [M − PF_6_]^+^ calcd. 997.3545; found 997.3557. LRMS (ESI^−^) *m*/*z*: 145.0 [PF_6_]^−^. IR (ATR, cm^−1^): 3029 (w), 2920 (w), 1602 (w), 1496 (w), 1486 (w), 1025 (m). Anal. calcd. for C_58_H_48_N_4_AuPF_6_ (1142.96) in %: C, 60.95; H, 4.23; N, 4.90; F, 9.97. Found: C, 60.95; H, 4.17; N, 4.85; F, 9.56.

#### 3.2.6. Bis-[1,3-dibenzyl-4,5-diphenylimidazol-2-ylidene]gold(I) Tetrafluoroborate (**3b**)


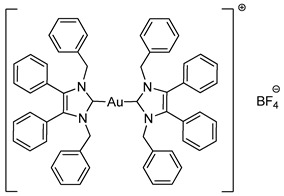


In a procedure identical to the above reaction: Imidazolium salt **6b** (45 mg, 0.093 mmol, 1.0 equiv.), Ag_2_O (23.6 mg, 0.102 mmol, 1.1 equiv.) and CH_2_Cl_2_-MeOH (1:1, 30 mL) for 5 h. (CH_3_)_2_SAuCl (14 mg, 0.046 mmol, 0.5 equiv.) was added and the reaction stirred for a further 48 h. After workup and precipitation a white solid was formed (44 mg, 87%). ^1^H-NMR (400 MHz, CDCl_3_, δ ppm): 7.28 (d, *J* = 7.4 Hz, 2H, ArCH), 7.18 (m, 20H, ArCH), 7.00 (d, *J* = 7.3 Hz, 8H, ArCH), 6.88 (d, *J* = 6.0 Hz, 8H, ArCH), 5.21 (s, 8H, CH_2_). ^13^C-NMR (101 MHz, CDCl_3_, δ ppm): 184.7 (NCN), 136.6 (C), 132.9 (C), 130.8 (CH), 129.6 (CH), 128.8 (CH), 127.9 (CH), 127.1 (CH), 126.8 (CH), 52.4 (CH_2_). ^19^F-NMR (376 MHz, CDCl_3_, δ ppm): −153.77, −153.82. HRMS (ESI^+^) *m*/*z*: [M − BF_4_]^+^ calcd. 997.3545; found 997.3514. LRMS (ESI^−^) *m*/*z*: 87.0 [BF_4_]^−^. IR (ATR, cm^−1^): 3061 (w), 3031 (w), 1604 (w), 1496 (m), 1449 (m), 1051 (s). Anal. calcd. for C_58_H_48_N_4_AuBF_4_ (1084.80) in %: C, 64.22; H, 4.46; N, 5.16; F, 7.01. Found: C, 63.92; H, 4.26; N, 5.12; F, 6.76.

#### 3.2.7. Triphenylphosphino-(1,3-dibenzyl-4,5-diphenylimidazol-2-ylidene)gold(I) Hexafluorophosphate (**4a**)


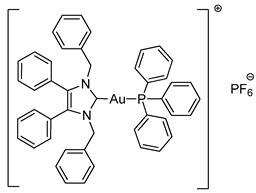


Using literature conditions [22], **2a** (63.3 mg, 0.10 mmol, 1.0 equiv.), PPh_3_ (34.1 mg, 0.13 mmol, 1.3 equiv.) and NaPF_6_ (20.2 mg, 0.12 mmol, 1.2 equiv.) were stirred in acetone (10 mL) at rt for 30 min. The solvent was removed under reduced pressure. The residue was dissolved in CH_2_Cl_2_ (15 mL) and filtered. The solvent was removed under reduced pressure to form a white solid. Hot ethanol (40 mL) was added to the solid and filtered under vacuum to give a white crystalline solid (77 mg, 76%). ^1^H-NMR (400 MHz, CDCl_3_, δ ppm): 7.53 (m, 3H, ArCH), 7.44 (td, *J* = 7.6, 1.9 Hz, 6H, ArCH), 7.30 (m, 17H, ArCH), 7.20 (m, 11H, ArCH), 7.05 (dd, *J* = 6.2, 3.1 Hz, 4H, ArCH), 5.41 (s, 4H, CH_2_). ^13^C-NMR (101 MHz, CDCl_3_, δ ppm): 136.8 (C), 134.3 (CH), 134.2 (CH), 133.1 (C), 132.2 (C), 130.9 (CH), 129.7 (CH), 129.6 (CH), 129.5 (CH), 129.0 (CH), 128.1 (CH), 127.1 (CH), 126.9 (C), 52.5 (CH_2_). ^19^F-NMR (376 MHz, CDCl_3_, δ ppm): −72.38, −74.28. ^31^P-NMR (162 MHz, CDCl_3_, δ ppm): 62.90, 39.86, −131.02, −135.43, −139.83, −144.24, −148.64, −153.05. HRMS (ESI^+^) *m*/*z*: [M − PF_6_]^+^ calcd. 859.2516; found 859.2479. LRMS (ESI^−^) *m*/*z*: 144.9 [PF_6_]^−^. IR (ATR, cm^−1^): 3053, 1585, 1496, 1434, 1025, 832. Anal. calcd. for C_47_H_39_N_2_AuP_2_F_6_ (1004.73) in %: C, 56.18; H, 3.91; N, 2.79; F, 11.35. Found: C, 55.95; H, 3.79; N, 2.68; F, 11.02.

#### 3.2.8. Triphenylphosphino-(1,3-dibenzyl-4,5-diphenylimidazol-2-ylidene)gold(I) Tetrafluoroborate (**4b**)


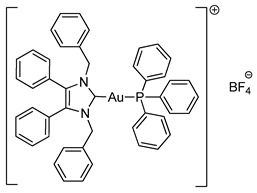


Using literature conditions [22], **2a** (63.3 mg, 0.10 mmol, 1.0 equiv.), PPh_3_ (34.1 mg, 0.13 mmol, 1.3 equiv.) and NaBF_4_ (13.2mg, 0.12 mmol, 1.2 equiv.) were stirred in acetone (10 mL) at rt for 30 min. The solvent was removed under reduced pressure. The residue was dissolved in CH_2_Cl_2_ (15 mL) and filtered. The solvent was removed under reduced pressure to form an oily residue. The residue was dissolved in hot ethanol (20 mL) and left to cool to form colourless crystals (30.6 mg, 32%). ^1^H-NMR (400 MHz, CDCl_3_, δ ppm): 7.52 (m, 3H, ArCH), 7.44 (m, 6H, ArCH), 7.31 (m, 9H, ArCH), 7.21 (m, 11H, ArCH), 7.06 (m, 4H, ArCH), 5.44 (s, 4H, CH_2_). ^13^C-NMR (101 MHz, CDCl_3_, δ ppm): 136.9 (C), 134.3 (CH), 134.2 (CH), 133.2 (C), 132.1 (C), 130.9 (CH), 129.8 (CH), 129.7 (CH), 129.6 (CH), 129.0 (CH), 128.1 (CH), 127.1 (CH), 126.9 (C), 52.6 (CH_2_). ^19^F-NMR (376 MHz, CDCl_3_, δ ppm): −153.73, −153.78. ^31^P-NMR (162 MHz, CDCl_3_, δ ppm): 25.21, −139.86. HRMS (ESI^+^) *m*/*z*: [M − BF_4_]^+^ calcd. 859.2511; found 859.2519. LRMS (ESI^−^) *m*/*z*: 87.1 [BF_4_]^−^. IR (ATR, cm^−1^): 3062, 1591, 1496, 1437, 1052, 692. Anal. calcd. for C_47_H_39_N_2_AuPBF_4_ (946.57) in %: C, 59.64; H, 4.15; N, 2.96; F, 8.03. Found: C, 59.52; H, 3.97; N, 2.90; F, 8.48.

#### 3.2.9. (1,3-Dibenzyl-4,5-diphenylimidazol-2-ylidene)(ethynyl)gold(I) (**5a**)


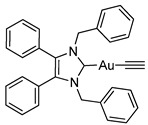


Ethynyltrimethylsilane (12 μL, 0.087 mmol, 1.1 equiv.) was added to a solution of **2a** (50 mg, 0.079 mmol, 1.0 equiv.) in THF (10 mL). TBAF (1 M in THF—0.87 mL, 0.087 mmol, 1.1 equiv.) was added dropwise and the reaction stirred at rt for 48 h. The solvent was removed under reduced pressure. The residue was dissolved in EtOAc (20 mL) and filtered through a short silica plug. The solvent was reduced to approximately 2 mL and pentane (40 mL) was added to precipitate the product. This was filtered, washed with pentane (15 mL) and dried in vacuo to give a white solid (30.1 mg, 61%). ^1^H-NMR (400 MHz, CDCl_3_, δ ppm): 7.29 (d, *J* = 7.4 Hz, 1H, ArCH) 7.20 (m, 10H, ArCH), 7.01 (dd, *J* = 7.4, 2.0 Hz, 4H, ArCH), 6.93 (m, 4H, ArCH), 5.45 (s, 4H, CH_2_), 1.75 (s, 1H, CH). ^13^C-NMR (101 MHz, CDCl_3_, δ ppm): 187.1 (NCN), 136.0 (C), 132.3 (C), 130.9 (CH), 129.4 (CH), 128.7 (CH), 128.6 (CH), 128.1 (CH), 127.7 (C), 127.6 (CH), 122.2 (C≡C), 90.7 (CH), 52.8 (CH_2_). HRMS (ESI^+^) *m*/*z*: [M + H]^+^ calcd. 623.1762; found 623.1749. IR (ATR, cm^−1^): 3291 (w) (C-H), 3053 (w), 3027 (w), 1977 (w) (C≡C), 1603 (w), 1448 (m), 1407 (m), 1023 (m). Anal. calcd. for C_31_H_25_N_2_Au (622.51) in %: C, 59.81; H, 4.05; N, 4.50. Found: C, 59.28; H, 3.95; N, 4.32.

#### 3.2.10. (1,3-Dibenzyl-4,5-diphenylimidazol-2-ylidene)(phenylethynyl)gold(I) (**5b**)


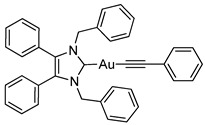


In a procedure identical to above reaction: (Phenylethynyl)trimethylsilane (17.0 μL, 0.087 mmol, 1.1 equiv.), **2a** (50 mg, 0.079 mmol, 1.0 equiv.) and TBAF (1 M in THF- 0.087 mL, 0.087 mmol, 1.1 equiv.) were stirred in THF (10 mL) at rt for 30 h. After workup and precipitation a white solid was formed (46.2 mg, 76%). ^1^H-NMR (400 MHz, CDCl_3_, δ ppm): 7.49 (d, *J* = 7.0 Hz, 2H, ArCH), 7.29 (m, 1H, ArCH), 7.19 (m, 12H, ArCH), 7.03 (d, *J* = 3.5 Hz, 4H, ArCH), 6.93 (m, 4H, ArCH), 5.49 (CH_2_). ^13^C-NMR (101 MHz, CDCl_3_, δ ppm): 187.4 (NCN), 136.1 (C), 132.5 (CH), 132.3 (C), 130.9 (CH), 129.3 (CH), 128.7 (CH), 128.6 (CH), 128.0 (CH), 127.9 (CH), 127.7 (C), 126.4 (CH), 125.8 (C≡C), 105.3 (C≡C), 52.9 (CH_2_). HRMS (ESI^+^) *m*/*z*: [M + H]^+^ calcd. 699.2075; found 699.2084. IR (ATR, cm^−1^): 3055 (w), 2947 (w), 2120 (w) (C≡C), 1593 (w), 1485 (m), 1444 (m), 1021 (m). Anal. calcd. for C_37_H_29_N_2_Au (698.60) in %: C, 63.61; H, 4.18; N, 4.01. Found: C, 63.26; H, 4.04; N, 3.95.

#### 3.2.11. (1,3-Dibenzyl-4,5-diphenylimidazol-2-ylidene)(4-methoxyphenylethynyl)gold(I) (**5c**)


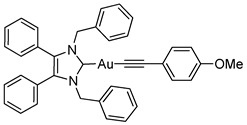


In a procedure identical to above reaction: (4-Methoxyphenylethynyl)trimethylsilane (18.5 μL, 0.087 mmol, 1.1 equiv.), **2a** (50 mg, 0.079 mmol, 1.0 equiv.) and TBAF (1 M in THF- 0.087 mL, 0.087 mmol, 1.1 equiv.) were stirred in THF (10 mL) at rt for 30 h. After workup and precipitation a white solid was formed (50 mg, 88%). ^1^H-NMR (400 MHz, CDCl_3_, δ ppm): 7.43 (d, *J* = 8.8 Hz, 2H, ArCH), 7.28 (m, 1H, ArCH), 7.19 (m, 10H, ArCH), 7.03 (d, *J* = 3.7 Hz, 4H, ArCH), 6.93 (d, *J* = 7.1 Hz, 4H, ArCH), 6.76 (d, *J* = 8.7 Hz, 2H, ArCH), 5.49 (s, 4H, CH_2_), 3.77 (s, 3H, CH_3_). ^13^C-NMR (101 MHz, CDCl_3_, δ ppm): 187.6 (NCN), 158.3 (C), 136.1 (C), 133.7 (CH), 132.3 (C), 130.9 (CH), 129.3 (CH), 128.6 (CH), 128.5 (CH), 128.0 (CH), 127.9 (CH), 125.9 (C≡C), 118.1 (C), 113.5 (CH), 105.1 (C≡C), 55.3 (CH_3_), 52.9 (CH_2_). HRMS (ESI^+^) *m*/*z*: [M + H]^+^ calcd. 729.2180; found 729.2171. IR (ATR, cm^−1^): 3030 (w), 2961 (w), 2831 (w) (OMe), 2111 (w) (C≡C), 1601 (m), 1503 (s), 1435 (m), 1023 (m). Anal. calcd. for C_38_H_31_N_2_OAu (728.63) in %: C, 62.64; H, 4.29; N, 3.84. Found: C, 62.42; H, 3.97; N, 3.58.

#### 3.2.12. (1,3-Dibenzyl-4,5-diphenylimidazol-2-ylidene)(4-fluorophenylethynyl)gold(I) (**5d**)


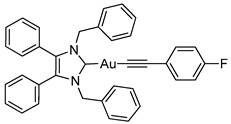


In a procedure identical to above reaction: (4-Fluorophenylethynyl)trimethylsilane (17.6 μL, 0.087 mmol, 1.1 equiv.), **2a** (50 mg, 0.079 mmol, 1.0 equiv.) and TBAF (1 M in THF- 0.087 mL, 0.087 mmol, 1.1 equiv.) were stirred in THF (10 mL) at rt for 30 h. After workup and precipitation a white solid was formed (39.6 mg, 70%). ^1^H-NMR (400 MHz, CDCl_3_, δ ppm): 7.45 (dd, *J* = 8.7, 5.5 Hz, 2H, CH), 7.29 (m, 1H, ArCH), 7.20 (m, 10H, ArCH), 7.02 (m, 5H, ArCH), 6.92 (m, 6H, ArCH), 5.49 (s, 4H, CH_2_). ^13^C-NMR (101 MHz, CDCl_3_, δ ppm): 187.3 (NCN), 136.1 (C), 134.1 (C), 134.0 (CH), 132.3 (C), 130.9 (CH), 129.3 (CH), 128.7 (CH), 128.6 (CH), 128.1 (CH), 127.7 (CH), 127.6 (CH), 121.8 (C≡C), 115.1 (C), 114.9 (CH), 104.1 (C≡C), 52.9 (CH_2_). ^19^F-NMR (376 MHz, CDCl_3_, δ ppm): −114. 32. HRMS (ESI^+^) *m*/*z*: [M + H]^+^ calcd. 717.1980; found 717.1978. IR (ATR, cm^−1^): 3054 (w), 2964 (w), 2113 (w) (C≡C), 1593 (w), 1497 (s), 1441 (m), 1203 (m) (C-F), 1022 (m). Anal. calcd. for C_37_H_28_N_2_FAu (716.60) in %: C, 62.01; H, 3.94; N, 3.91; F, 2.65. Found: C, 61.68; H, 3.77; N, 3.83; F, 3.04.

#### 3.2.13. (1,3-Dibenzyl-4,5-diphenylimidazol-2-ylidene)[4-(trifluoromethyl)phenylethynyl]gold(I) (**5e**)


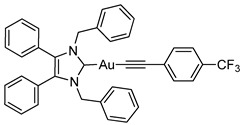


In a procedure identical to above reaction: [4-(Trifluoromethyl)phenyl](trimethylsilyl)acetylene (20.4 μL, 0.087 mmol, 1.1 equiv.), **2a** (50 mg, 0.079 mmol, 1.0 equiv.) and TBAF (1 M in THF—0.087 mL, 0.087 mmol, 1.1 equiv.) were stirred in THF (10 mL) at rt for 24 h. After workup and precipitation a white solid was formed (37 mg, 60%). ^1^H-NMR (400 MHz, CDCl_3_, δ ppm): 7.56 (d, *J* = 8.0 Hz, 2H, ArCH), 7.46 (d, *J* = 8.3 Hz, 2H, ArCH), 7.29 (d, *J* = 7.5 Hz, 1H, ArCH), 7.21 (m, 10H, ArCH), 7.03 (dd, *J* = 7.2, 2.2 Hz, 4H, ArCH), 6.93 (m, 4H, ArCH), 5.49 (s, 4H, CH_2_). ^13^C-NMR (101 MHz, CDCl_3_, δ ppm): 187.0 (NCN), 136.0 (C), 132.5 (CH), 132.4 (C), 132.1 (C), 130.9 (CH), 129.4 (CH), 128.7 (CH), 128.6 (CH), 128.1 (CH), 127.6 (CH), 127.5 (C), 124.9 (CH), 124.8 (C≡C), 103.9 (C≡C), 52.9 (CH_2_). ^19^F-NMR (376 MHz, CDCl_3_, δ ppm): −62.52. HRMS (ESI^+^) *m*/*z*: [M + H]^+^ calcd. 767.1948; found 767.1922. IR (ATR, cm^−1^): 3024 (w), 2119 (w) (C≡C), 1608 (m), 1493 (w), 1434 (m), 1113 (s) (C-F), 1063 (s). Anal. calcd. for C_38_H_28_N_2_F_3_Au (766.60) in %: C, 59.54; H, 3.68; N, 3.65; F, 7.43. Found: C, 59.33; H, 3.43; N, 3.52; F, 7.09.

### 3.3. Structure Determination

Crystal data were collected using a Rigaku Oxford Diffraction (former Agilent Technologies, former Oxford Diffraction, Chalgrove, Oxfordshire, UK) SuperNova A diffractometer. Compounds **2b**, **2c** and **5a** were measured with Mo-K_α_ (0.71073 Å), all others with Cu-K_α_ (1.54184 Å) radiation. A complete dataset was collected, assuming that the Friedel pairs were not equivalent. An analytical absorption correction based on the shape of the crystal was performed [31]. The structures were solved by direct methods using SHELXS-2014 (SHELX, Göttingen, Germany) and refined by full matrix least-squares on F2 for all data using SHELXL-2014 [32]. Hydrogen atoms were added at calculated positions and refined using a riding model. Their isotropic temperature factors were fixed to 1.2 times (1.5 times for methyl groups) the equivalent isotropic displacement parameters of the carbon atom the H-atom was attached to. Anisotropic thermal displacement parameters were used for all non-hydrogen atoms. CCDC 2012885–2012893, 2013114 and 2013115 contain the supplementary crystallographic data for this paper. This data can be obtained free of charge from https://www.ccdc.cam.ac.uk/structures/.

### 3.4. Biological Evaluation

#### 3.4.1. Cell Culture Conditions and Stock Solutions

The HCT-116^wt^ (DSMZ ACC-581) colon carcinoma cells and the MCF-7^topo^ (DMSZ ACC-115) multidrug-resistant human breast carcinoma cells were cultivated in Dulbecco′s Modified Eagle Medium (Gibco, ThermoFisher, Waltham, MA, USA), supplemented with 10% fetal bovine serum (Biochrom) and 1% Antibiotic-Antimycotic (Gibco, ThermoFisher, Waltham, MA, USA) at 37 °C, 95% humidity and 5% CO_2_. If not indicated otherwise, all incubation steps of the assay were conducted under these cell culture conditions. The gold complexes used in the MTT assay were dissolved in DMF (10 mM). These solutions were diluted with sterile Milli-Q water as appropriate. This caused precipitation which was treated with 4.44 µL/mL tween80 (Sigma) to produce soluble solutions of the complexes that were used right away.

#### 3.4.2. Anti-Proliferative Activity (MTT-assay)

The complexes **2a**–**5e** were investigated for their anti-proliferative effect on human colon carcinoma cell line HCT-116^wt^ and the multidrug-resistant human breast carcinoma cell line MCF-7^topo^ via the MTT (Glentham Life Sciences)-based proliferation assay. Cells were seeded at 0.05 × 10^6^ cells per mL (cpm) into the wells of 96-well microtiter plates (100 µL/well) and incubated for 24 h. Appropriate dilutions in H_2_O of the complexes or equal amounts of the solvent (negative controls) were added into the wells and the cells were further incubated for 72 h. Before staining of the viable cells the plates were centrifuged (300 g, 5 min, 4 °C) and the back medium was discarded. A total of 50 µL of a 0.05% MTT solution (PBS) was added to each well. After another 2 h of incubation the plates were centrifuged as before and the MTT solution was discarded again. To dissolve the cells and the formed violet water-insoluble formazan, 25 µL of a SDS/DMSO solution (10%, 0.6% AcOH) was added to each well and plates were further incubated for at least 1 h. The absorbances at 570 nm (formazan) and at 630 nm (background) were measured. The absorbance of formazan is directly linked to the amount of metabolically active (viable) cells in the wells. The absorbance of the wells treated with the solvent was set to 100% viable cells, and the percentage of viable cells in the wells treated with the gold complexes was calculated accordingly. IC_50_ values were determined using Graphpad Prism, means and SD were calculated from four independent experiments.

## 4. Conclusions

NHC-gold(I) complexes were synthesised and studied for their biological properties in order to develop potential anticancer drugs. Complexes of the type NHC*-Au(I)-L (L = halides and alkynes) and [NHC*-Au-L]^+^ (L = carbenes and phosphines) were synthesised. All complexes are novel, thus were fully characterised, including with X-ray crystallography. Aurophilic interactions were observed in complexes **2b**, **2c**, **5a**, **5d** and **5e**, with Au···Au distances of 3.2077(9)–3.5850(3) Å. Compound **5d** exhibited the shortest Au···Au contact with distances of 3.2077(9) Å between the gold centres. The in vitro anticancer activity of these gold(I)-based complexes were tested against the human colon cancer cell line HCT-116^wt^ and the human breast cancer cell line MCF-7^topo^. The acetylide-based complexes displayed the lowest activity, followed by the phosphines. Outstanding activity was found with the biscarbene complexes, suggesting that not only are NHC ligands more favourable than phosphines but also that two NHCs render highly active complexes. The iodide derivative **2c** showed excellent cytotoxicity against both cell lines, reaching sub-micromolar activity against HCT-116^wt^. Overall, the cationic biscarbenes **3a** and **3b** presented the highest cytotoxic response with sub-micromolar IC_50_ values against both cell lines; the best observed activity was on the colon cancer cell line HCT-116^wt^ with an IC_50_ value of 0.29 ± 0.01 μM. These results are very promising and encourage further biological studies to assess their capabilities as anticancer drug candidates.

## Figures and Tables

**Figure 3:**
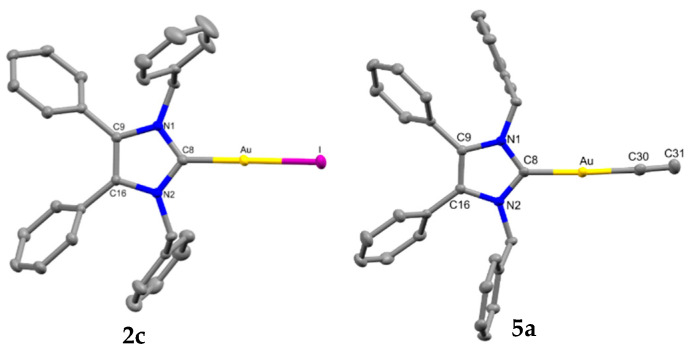
X-ray diffraction structures of NHC*-Au-I (**2c**) and NHC*-Au-CCH (**5a**); thermal ellipsoids drawn on the 50% probability level. Solvent molecules and hydrogen atoms have been omitted for clarity.

**Figure 4:**
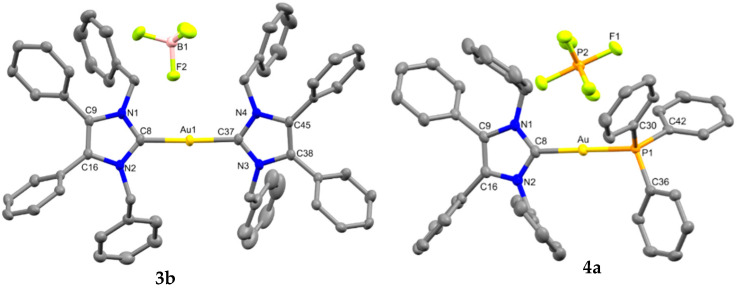
X-ray diffraction structures of [NHC*_2_Au]BF_4_ (**3b**) and [NHC*-Au-PPh_3_]PF_6_ (**4a**); thermal ellipsoids drawn on the 50% probability level. Hydrogen atoms have been omitted for clarity.

**Table 1:** Selected bond lengths (Å) and angles (°) of complexes **2b**–**5e**.

	C(8)-Au	Au-X	C(8)-Au-X	C(30)-C(31)	C(31)-C(32)	Au···Au Contact
2b ^a^	2.000(3)	2.4043(3)	177.379(8)	-	-	3.5850(3)
2c ^b^	2.0057(18)	2.55545(15)	174.66(5)	-	-	3.5449(3)
3a ^c^	2.022(2)	2.021(2)	176.14(8)	-	-	-
3b ^c^	2.022(3)	2.019(3)	173.92(10)	-	-	-
4a ^d^	2.038(2)	2.2808(5)	177.84(6)	-	-	-
4b ^d^	2.035(2)	2.2834(6)	179.40(6)	-	-	-
5a ^e^	2.018(2)	1.993(3)	175.94(9)	1.198(4)	-	3.5242(3)
5b ^e^	2.028(4)	1.994(4)	179.65(16)	1.196(6)	1.437(5)	-
5c ^e^	2.019(2)	1.994(2)	173.28(9)	1.197(4)	1.442(3)	-
5d ^e^	2.021(4)	1.982(5)	173.24(18)	1.217(7)	1.430(7)	3.2077(9) 3.2666(9)
5e ^e^	2.033(4)	1.999(4)	173.46(17)	1.193(6)	1.445(6)	3.3318(5)

^a^ X = Br. ^b^ X = I. ^c^ X = C(37). ^d^ X = P(1). ^e^ X = C(30).

**Table 2:** IC_50_ values (μM) of compounds **2a**–**5e** against HCT-116^wt^ and MCF-7^topo^ cells after 72 h of incubation.

Compound	HCT-116^wt^	MCF-7^topo^	Compound	HCT-116^wt^	MCF-7^topo^
Cisplatin ^a^	5.42 ± 0.12	18.21 ± 0.10	4a	1.3 ± 0.1	1.5 ± 0.2
Auranofin ^a^	3.78 ± 0.10	2.89 ± 0.05	4b	2.3 ± 0.2	2.4 ± 0.2
2a	22.7 ± 1.2	20.8 ± 1.9	5a	3.2 ± 0.3	7.3 ± 0.8
2b	16.7 ± 2.5	12.8 ± 2.4	5b	14.1 ± 1.3	>50
2c	0.64 ± 0.01	1.1 ± 0.2	5c	8.3 ± 0.5	>50
3a	0.41 ± 0.01	0.70 ± 0.06	5d	>50	>50
3b	0.29 ± 0.01	0.80 ± 0.04	5e	>50	>50

^a^ Cisplatin and auranofin IC_50_ values against HCT-116 and MCF-7 cell lines have been included for comparative purposes [30].

## Supplementary Materials

The following are available online, Figure S1–S26: ^1^H and ^13^C-NMR spectra, Tables S1–S3: Crystal data and structure refinement, Table S4: CCDC numbers, Figure S27–S34: X-ray diffraction structures for complexes **2b**, **3a**, **4b** and **5b**–**5e**.
