# Supplementary material for: Novel Anticancer NHC*-Gold(I) Complexes Inspired by Lepidiline A
Source: Molecules. 2020 Jul 30;25(15):3474. doi: 10.3390/molecules25153474 (PMC7436326; doi:10.3390/molecules25153474)
Supplement: Supplementary file 1 [file molecules-25-03474-s001.pdf]

Supplementary Information

Novel Anticancer NHC*-Gold(I) Complexes Inspired by Lepidiline A

Danielle Curran[1], Helge-Müller-Bunz[1], Sofia I. Bär[2], Rainer Schobert[2], Xiangming Zhu[1] and Matthias Tacke[1,*]

[1] School of Chemistry, University College Dublin, Belfield, Dublin 4, Ireland; danielle.curran@ucdconnect.ie (D.C.); helge.muellerbunz@ucd.ie (H.M.B.); xiangming.zhu@ucd.ie (X.Z.)

[2] Organic Chemistry Laboratory, University of Bayreuth, Universitätsstr. 30, 95440 Bayreuth, Germany; sofia.baer@uni-bayreuth.de (S.B.); rainer.schobert@uni-bayreuth.de (R.S.)

[*] Correspondence: matthias.tacke@ucd.ie; Tel.: +353-1-7168428 (M.T.)

Contents

^{1}H and ^{13}C-NMR spectra. **Figure S1-S26**

Crystal data and structure refinement. **Table S1-S3**

CCDC numbers for complexes **2b**-**5e**. **Table S4**

X-ray diffraction structures of complexes **2b**, **3a**, **4b** and **5b**-**5e**. **Figure S27-S34**

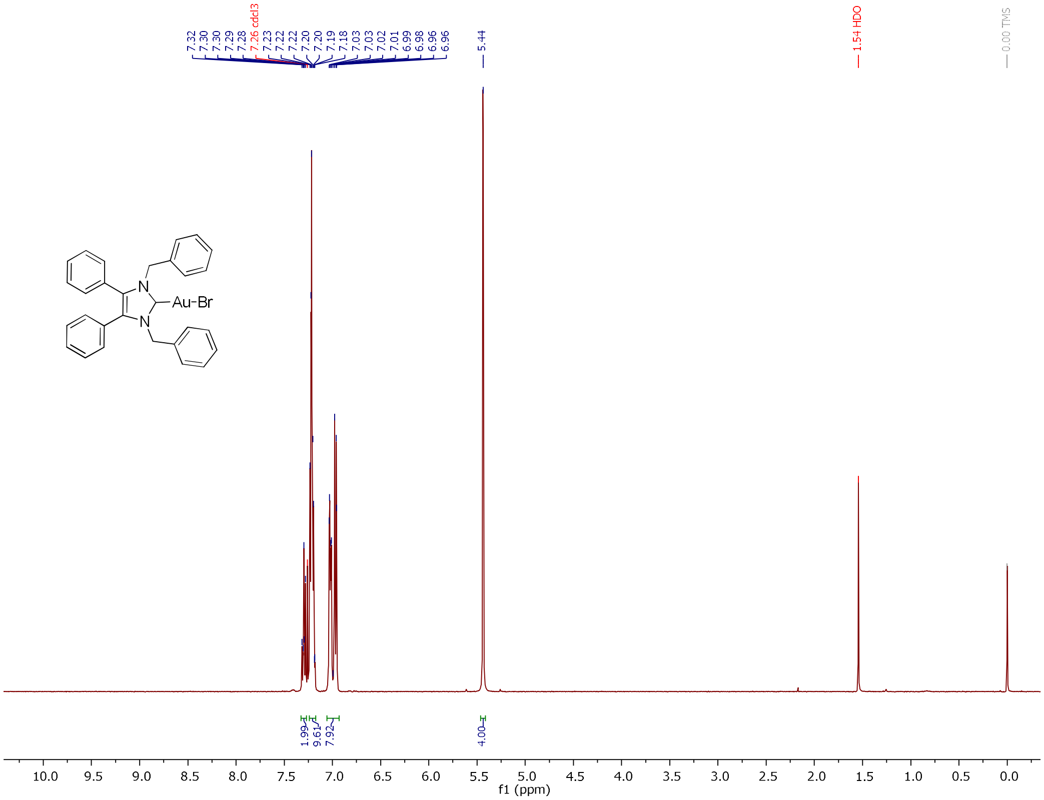

Figure S1. ^{1}H-NMR spectra of **2b** in $CDCl_3$.

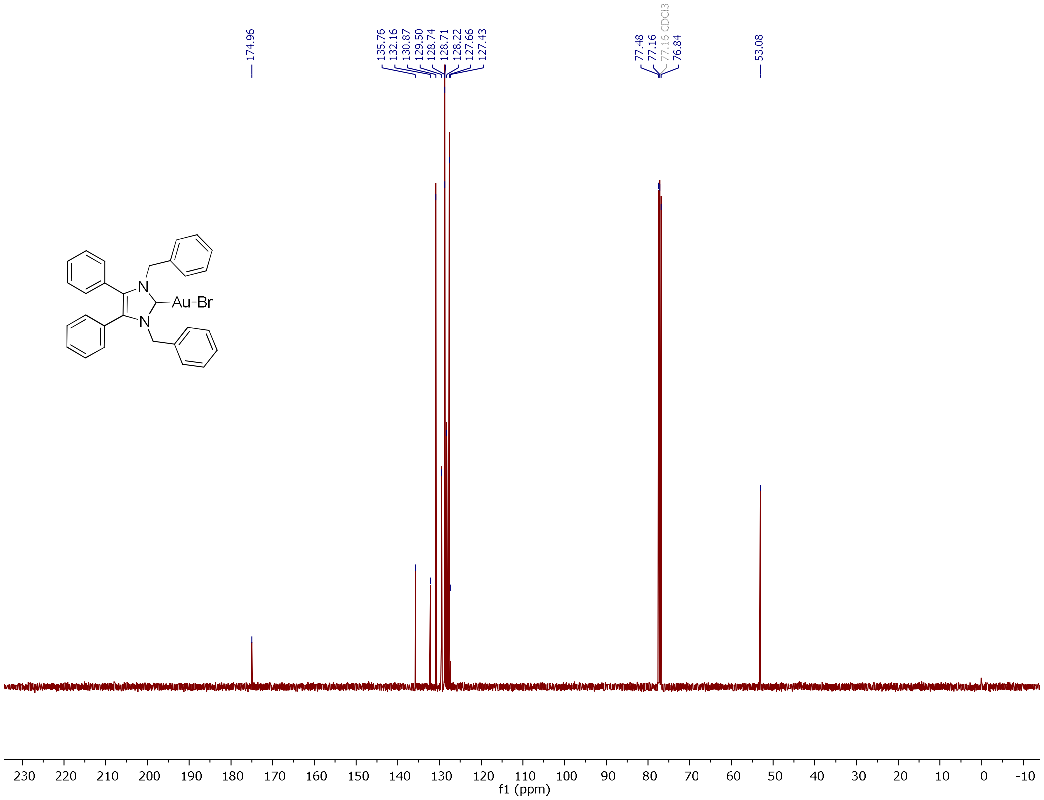

Figure S2. ^{13}C-NMR spectra of **2b** in $CDCl_3$.

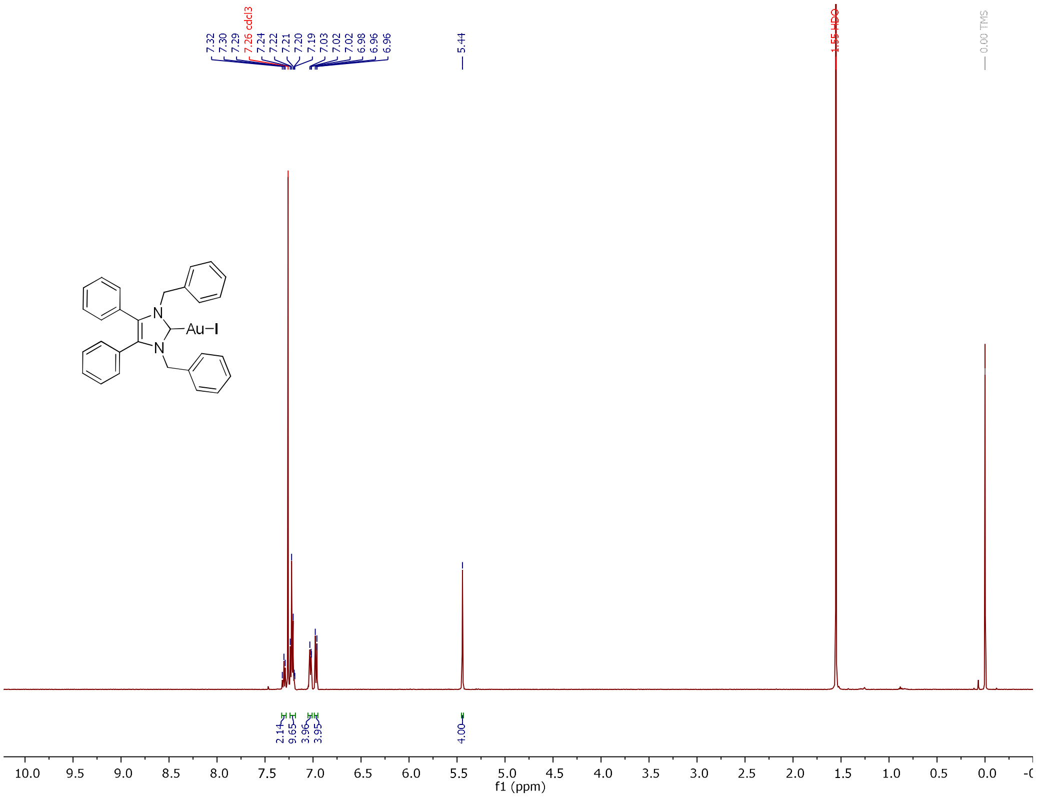

Figure S3. ^{1}H-NMR spectra of **2c** in $CDCl_3$.

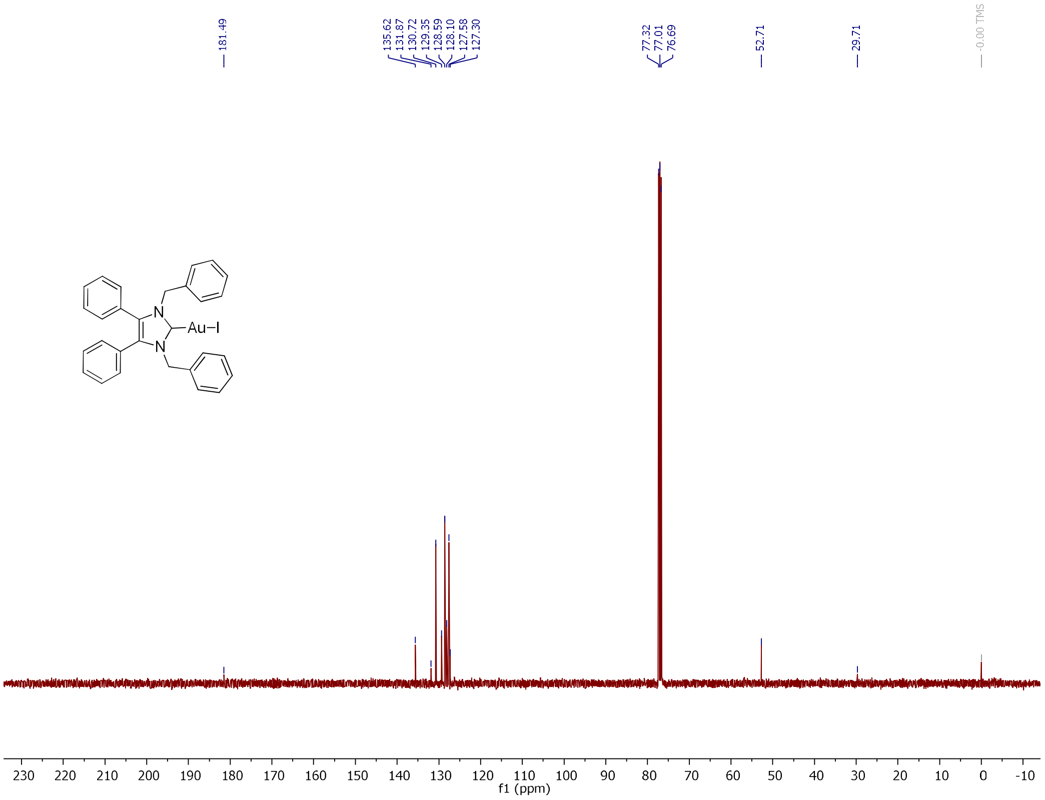

Figure S4. ^{13}C-NMR spectra of **2c** in $CDCl_3$.

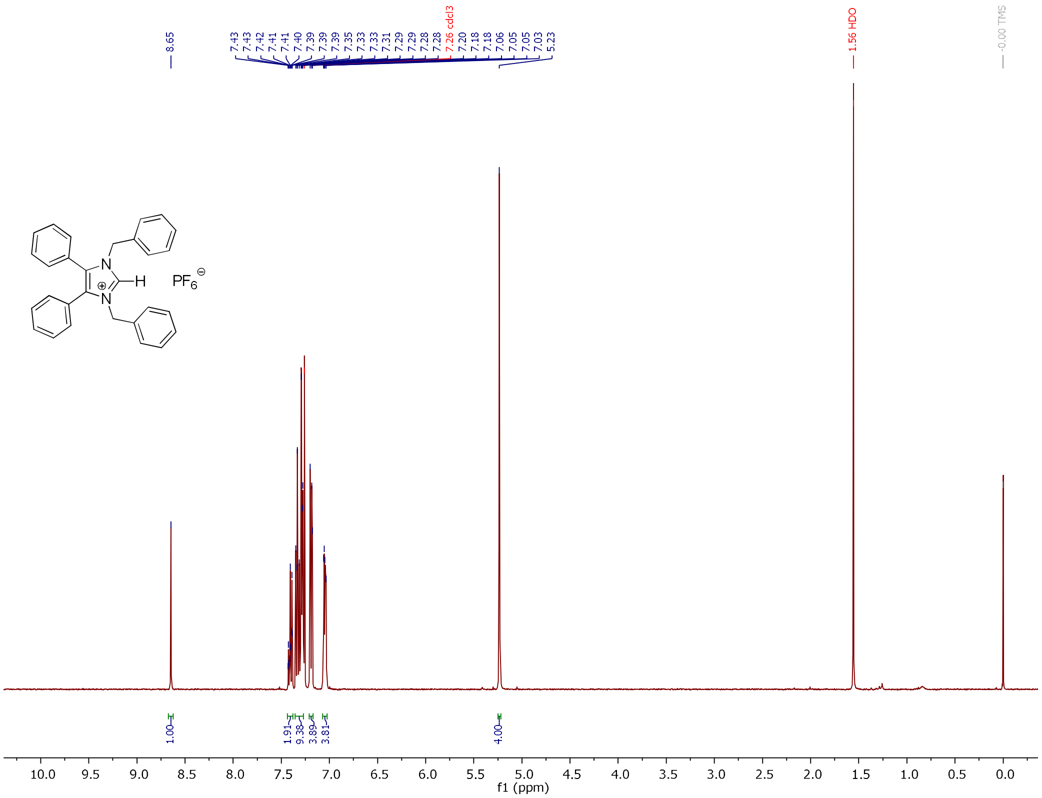

Figure S5. ^{1}H-NMR spectra of **6a** in $CDCl_3$.

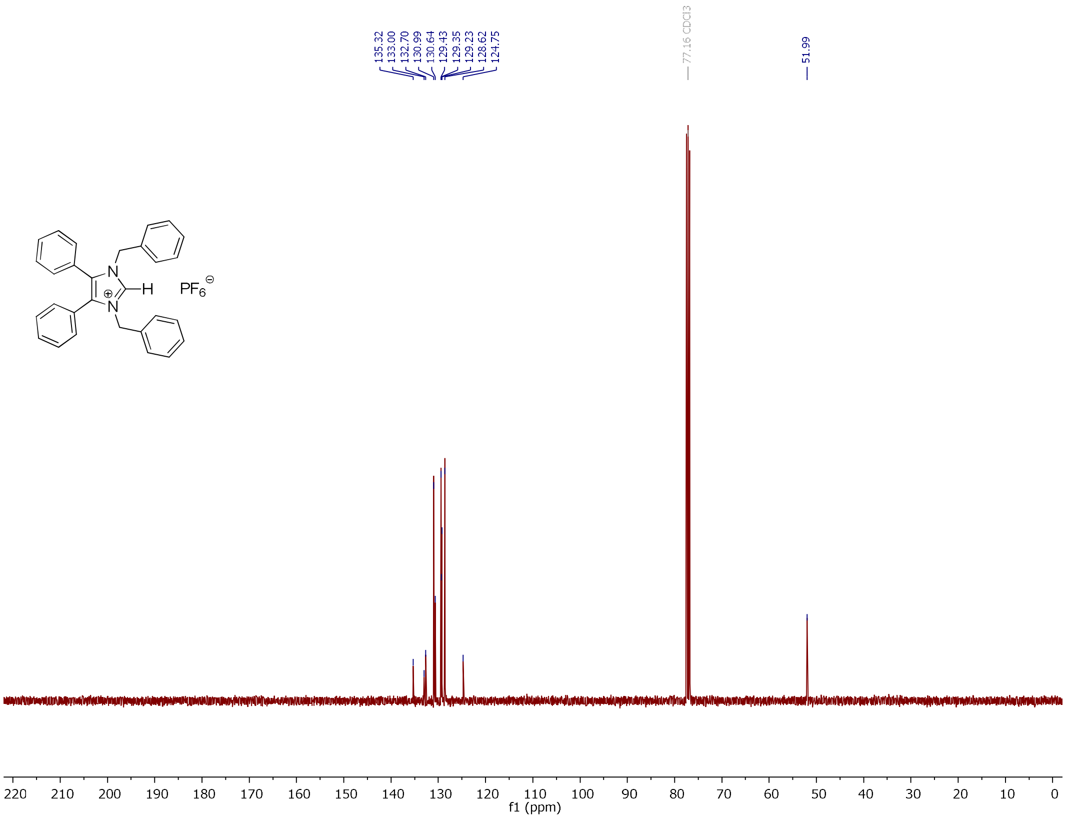

Figure S6. ^{13}C-NMR spectra of **6a** in $CDCl_3$.

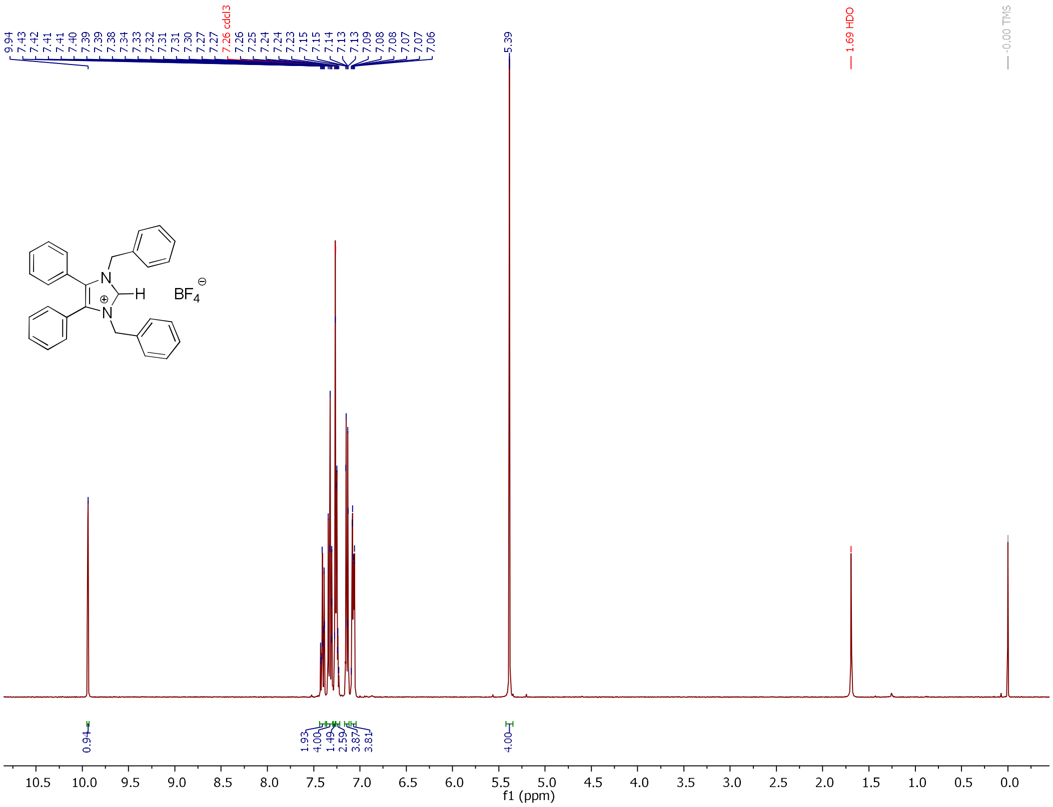

Figure S7. ^{1}H-NMR spectra of **6b** in $CDCl_3$.

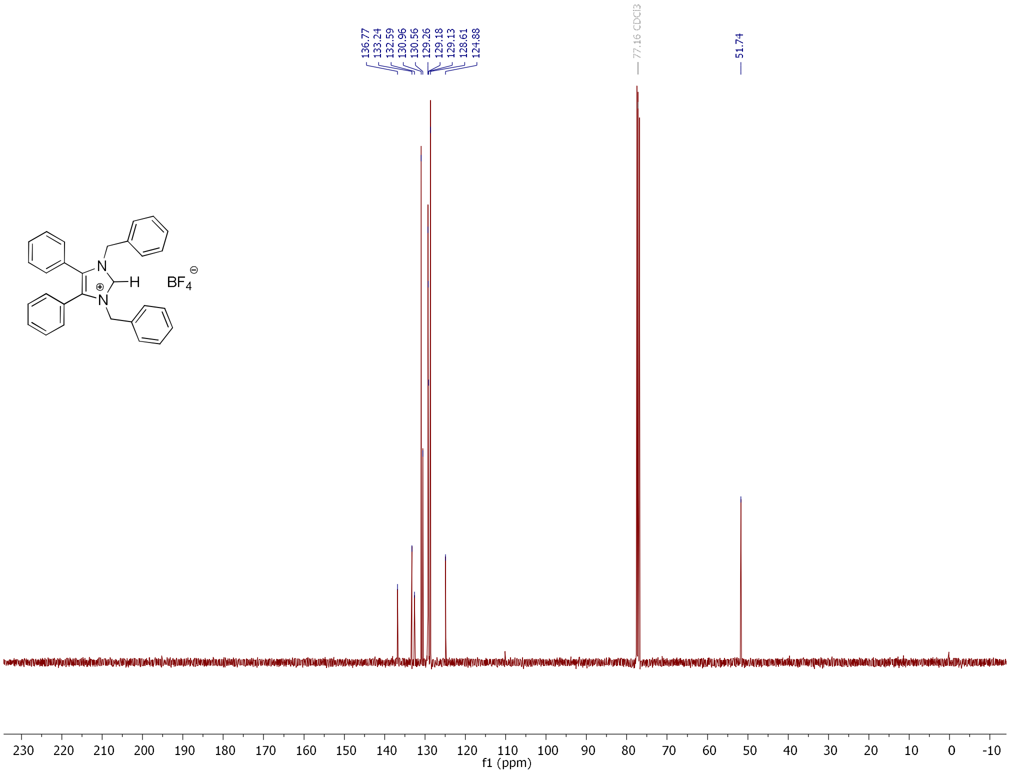

Figure S8. ^{13}C-NMR spectra of **6b** in $CDCl_3$.

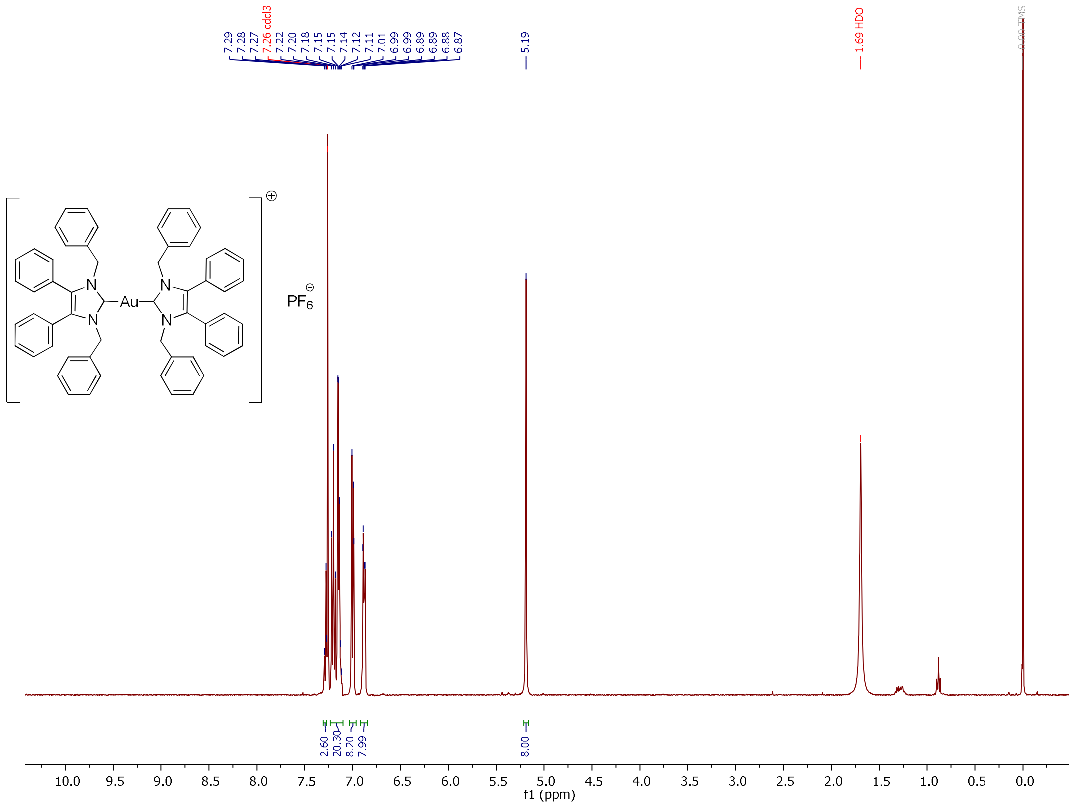

Figure S9. ^{1}H-NMR spectra of **3a** in $CDCl_3$.

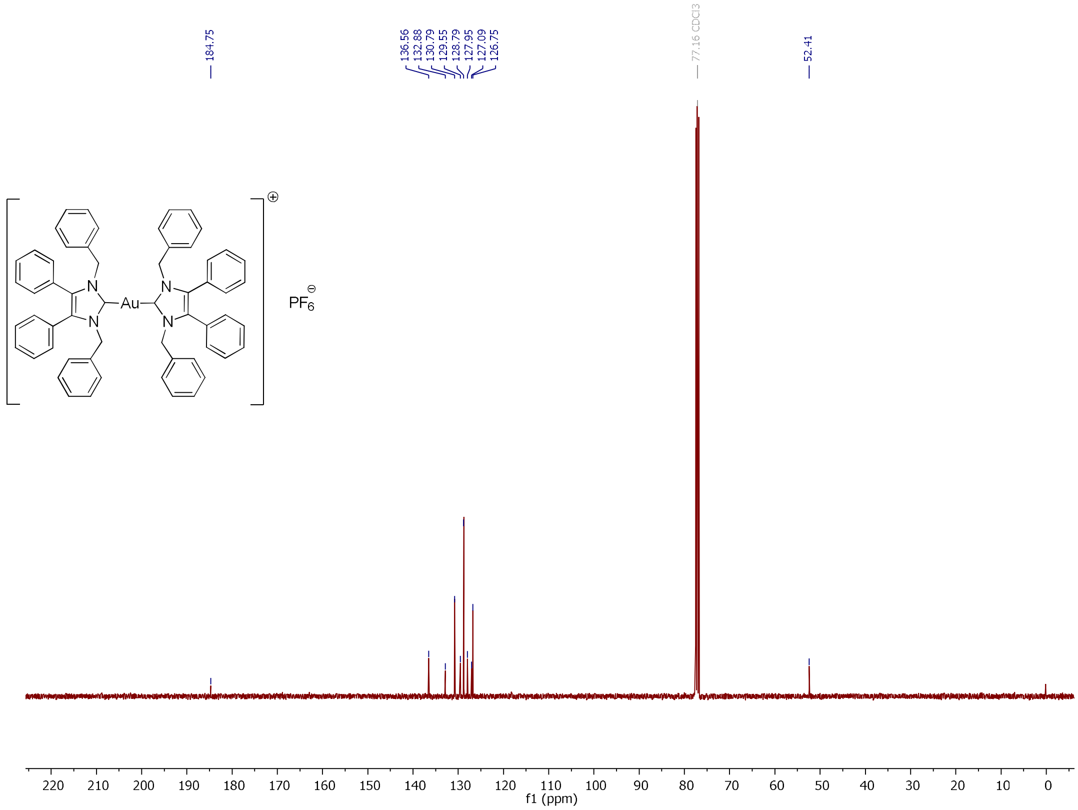

Figure S20. ^{13}C-NMR spectra of **3a** in $CDCl_3$.

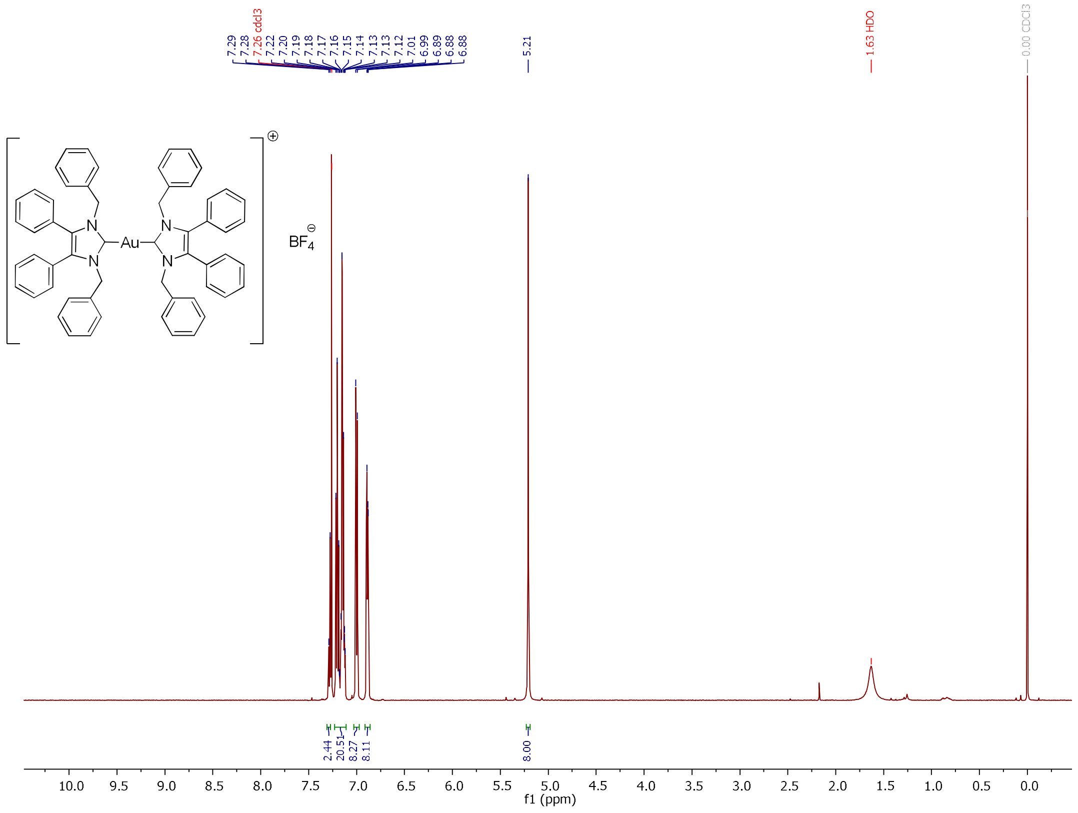

Figure S13. ^{1}H-NMR spectra of **3b** in $CDCl_3$.

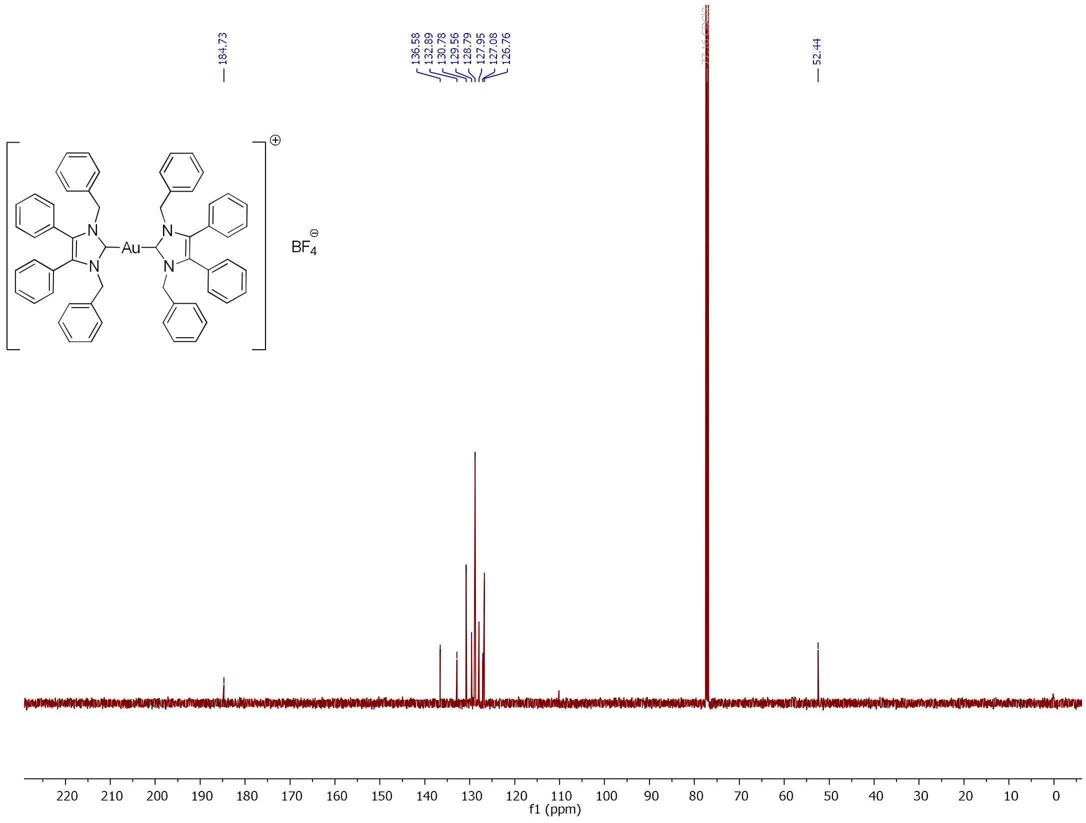

Figure S42. ^{13}C-NMR spectra of **3b** in $CDCl_3$.

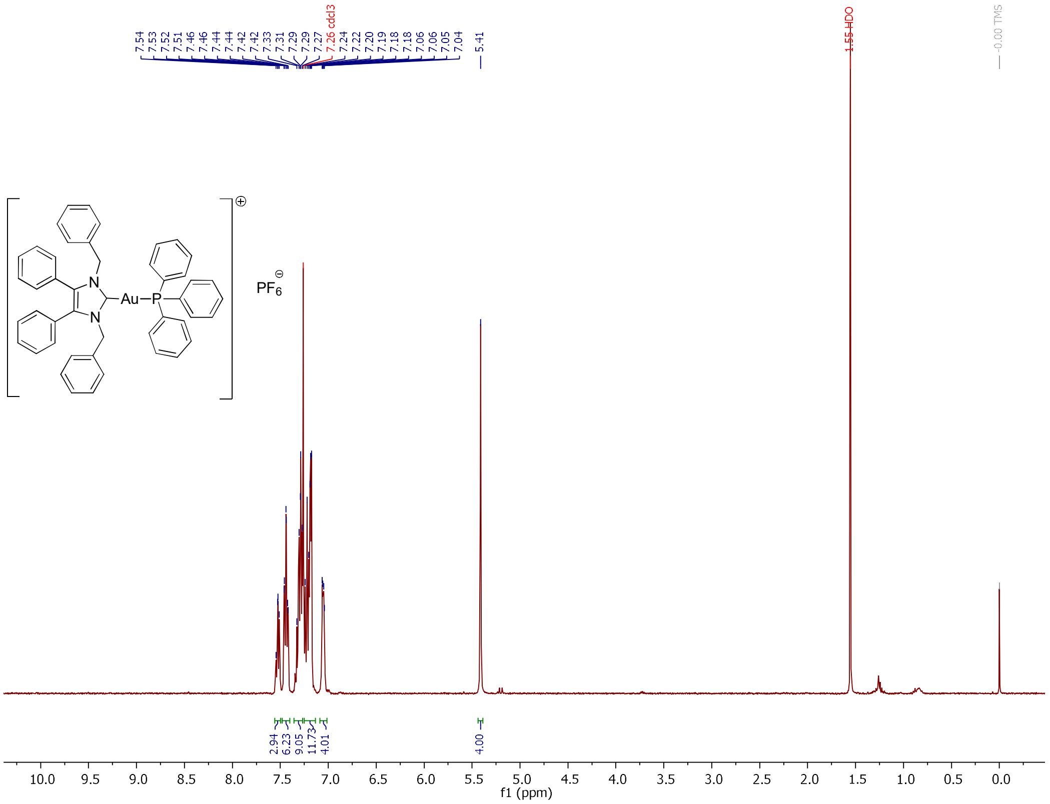

Figure S53. ^{1}H-NMR spectra of **4a** in $CDCl_3$.

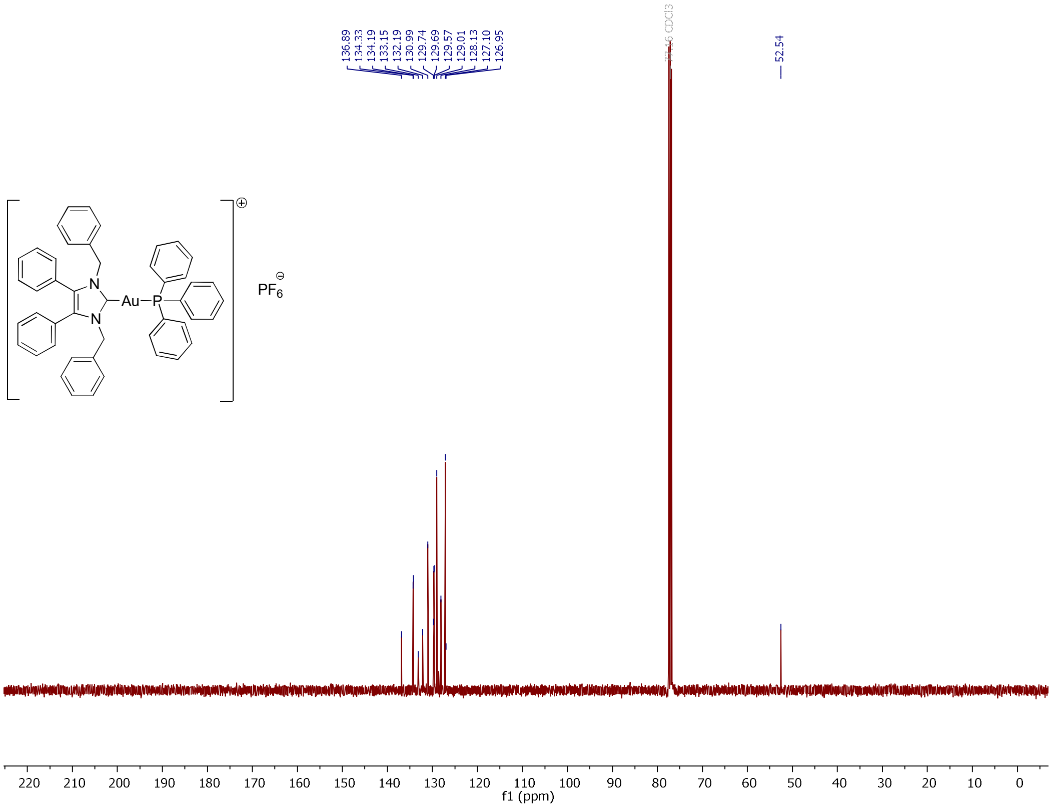

Figure S64. ^{13}C-NMR spectra of **4a** in $CDCl_3$.

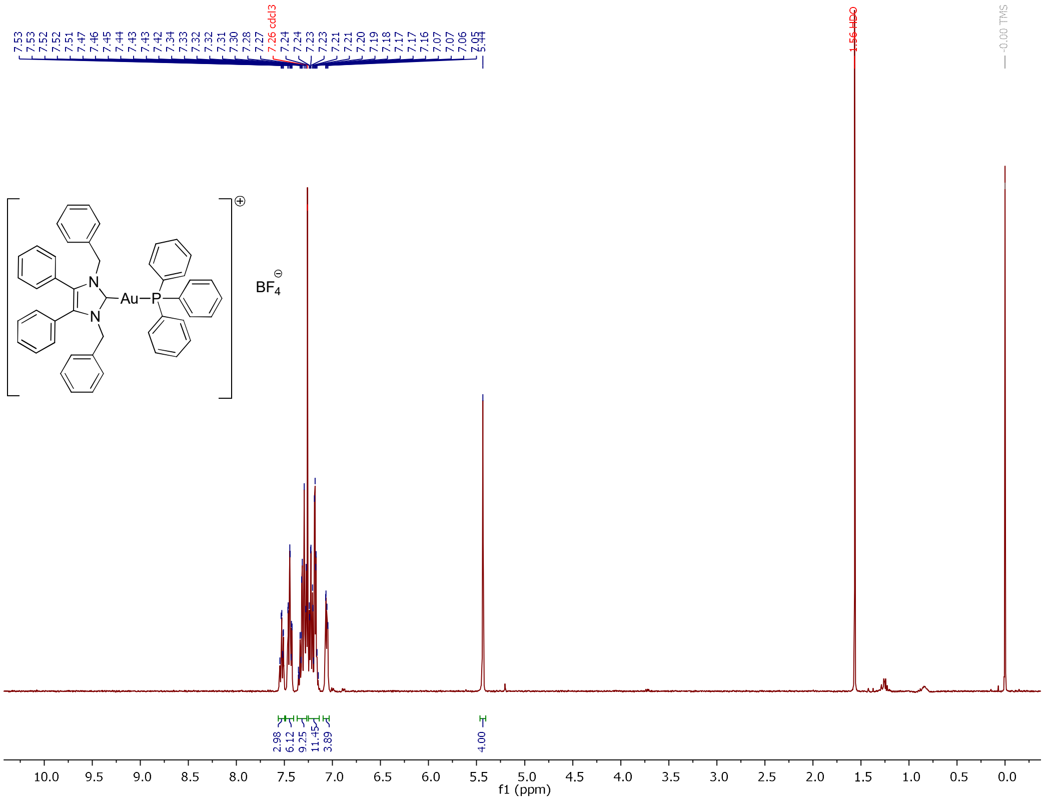

Figure S75. ^{1}H-NMR spectra of **4b** in $CDCl_3$.

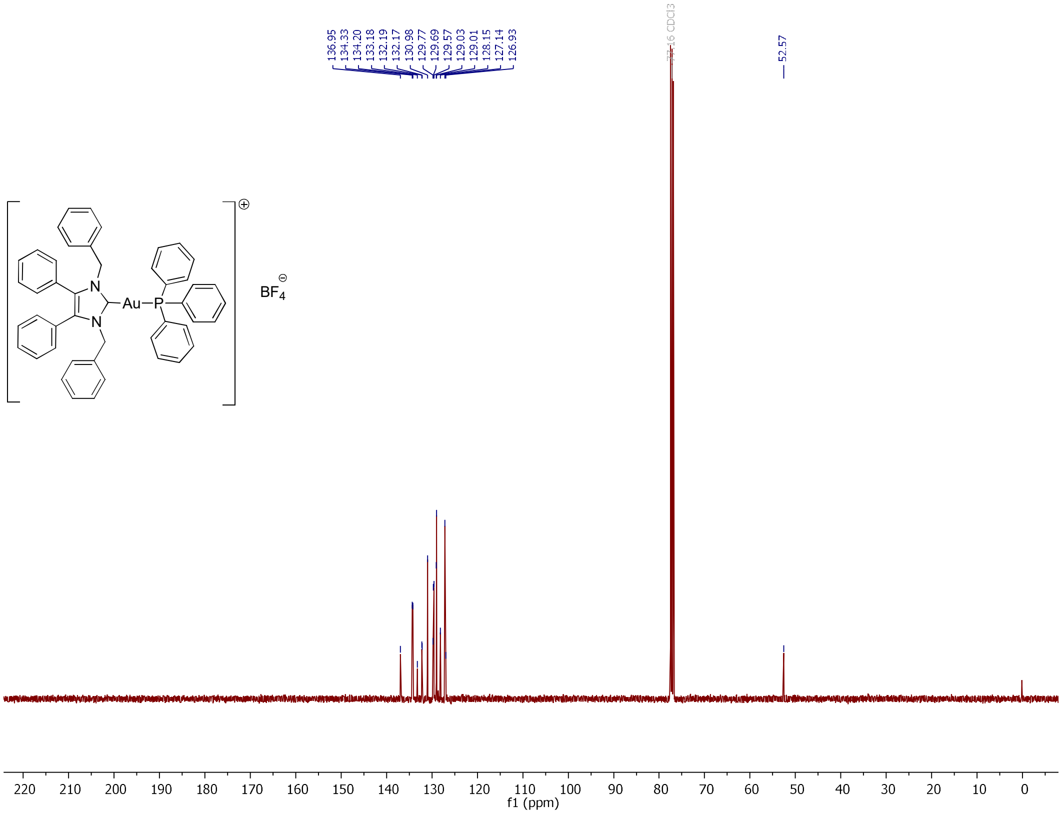

Figure S86. ^{13}C-NMR spectra of **4b** in $CDCl_3$.

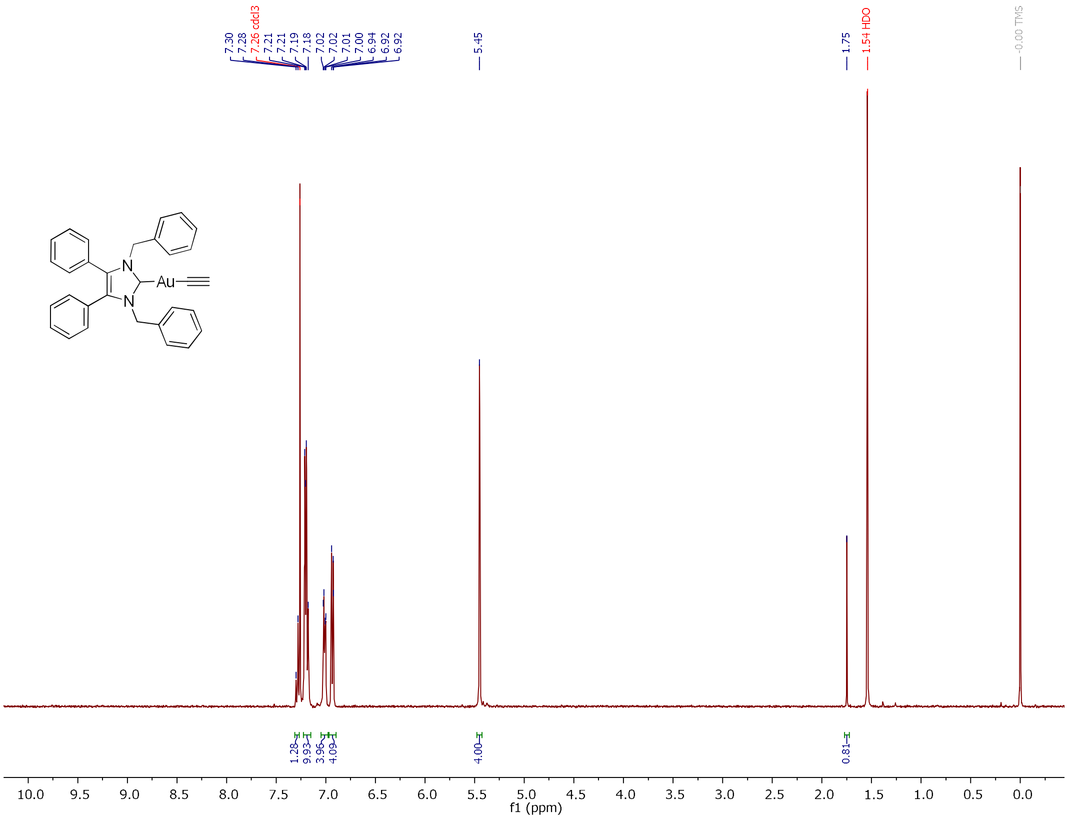

Figure S97. ^{1}H-NMR spectra of **5a** in $CDCl_3$.

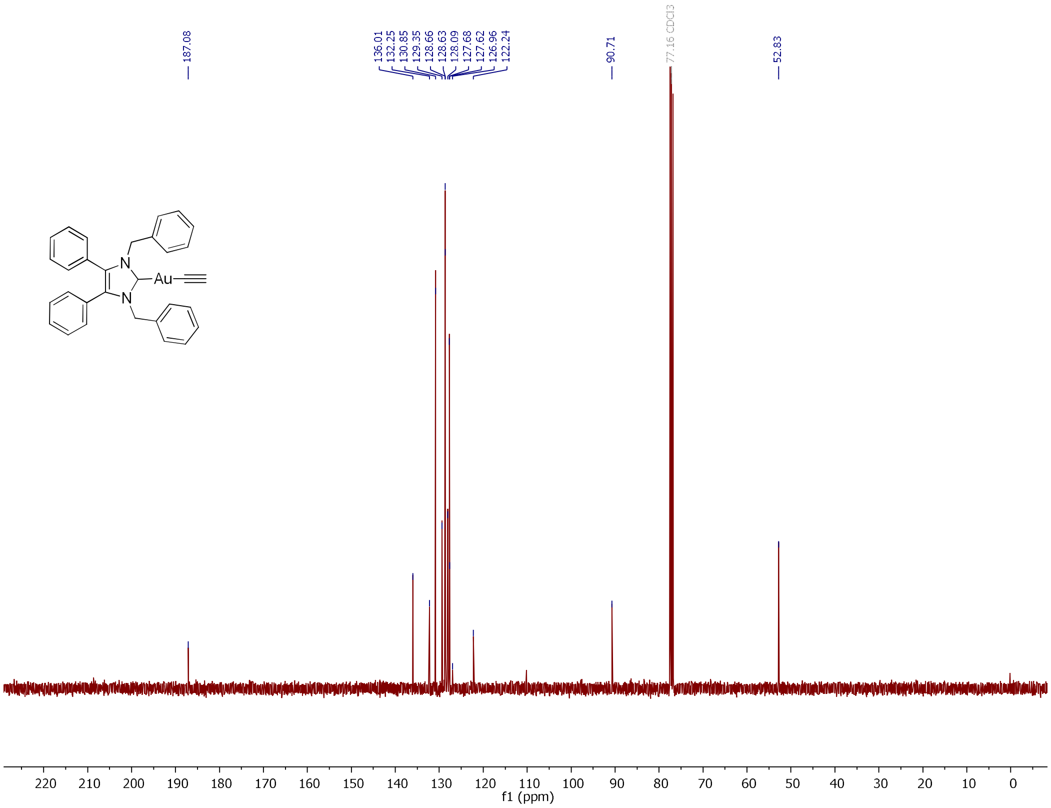

Figure S108. ^{13}C-NMR spectra of **5a** in $CDCl_3$.

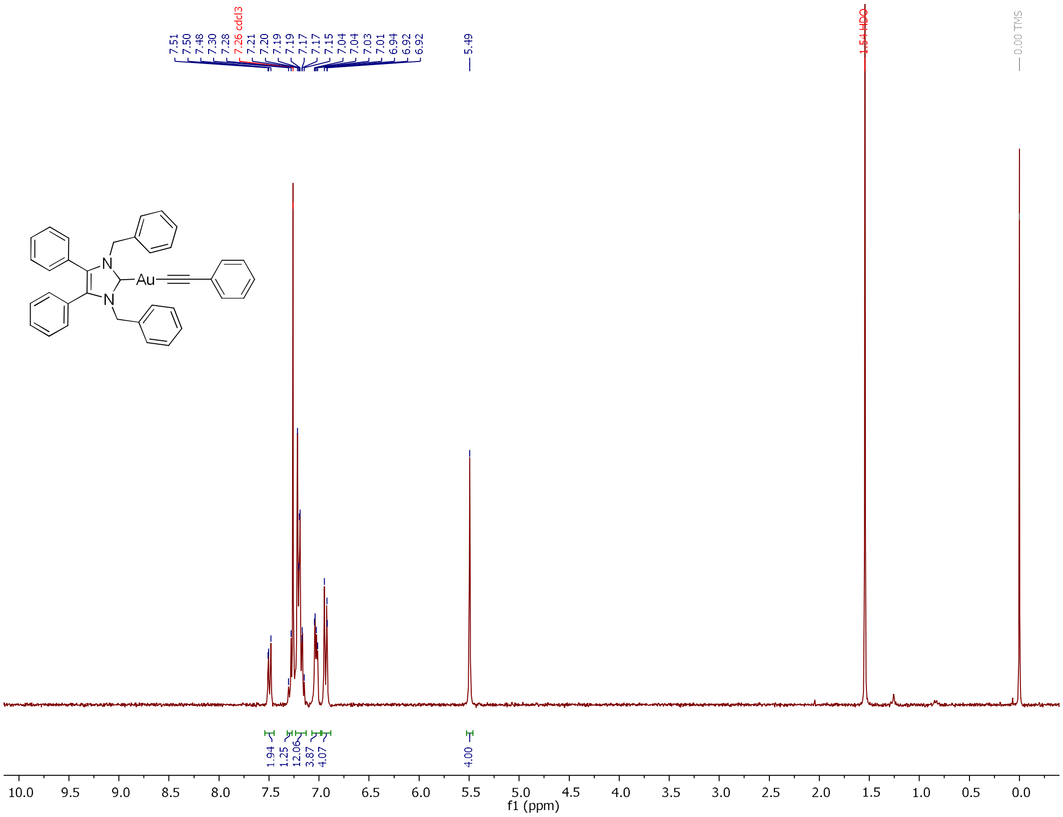

Figure S119. ^{1}H-NMR spectra of **5b** in $CDCl_3$.

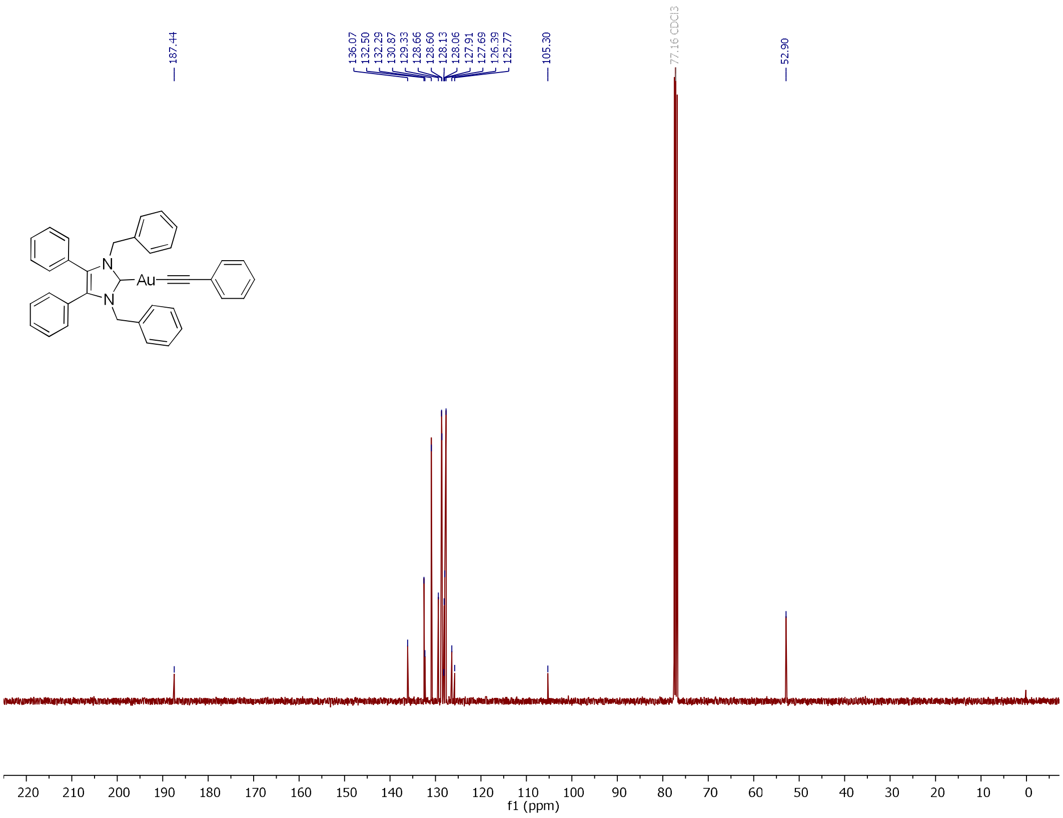

Figure S20. ^{13}C-NMR spectra of **5b** in $CDCl_3$.

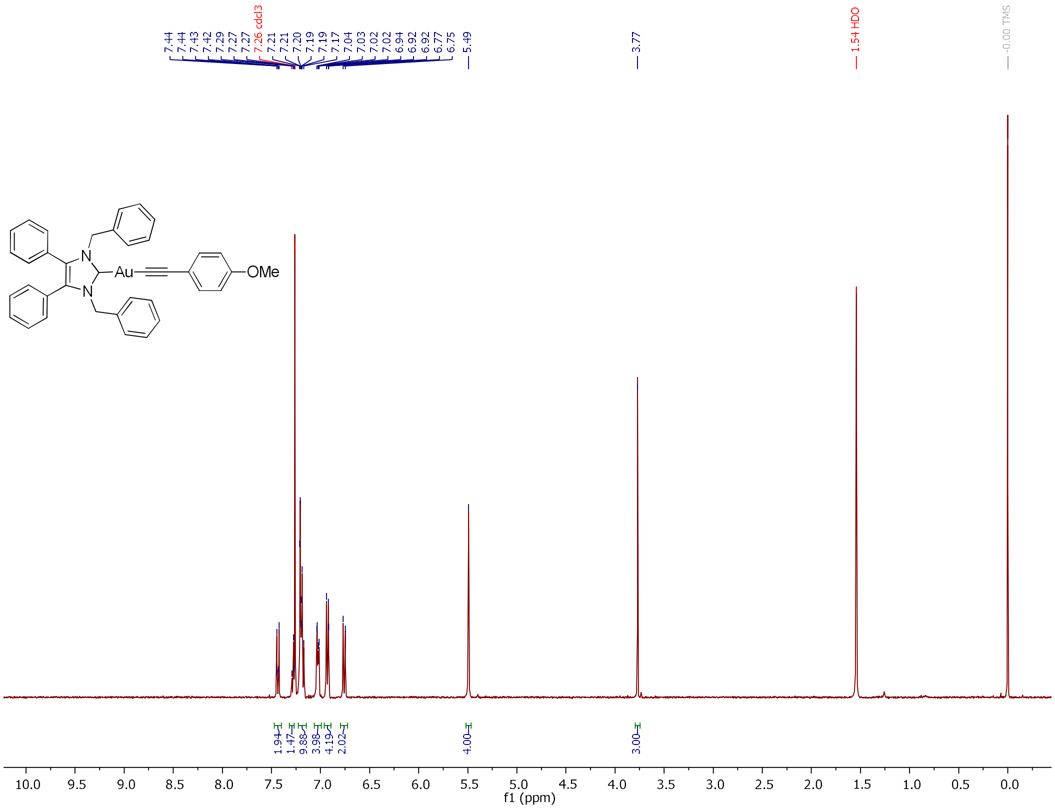

Figure S212. ^{1}H-NMR spectra of **5c** in $CDCl_3$.

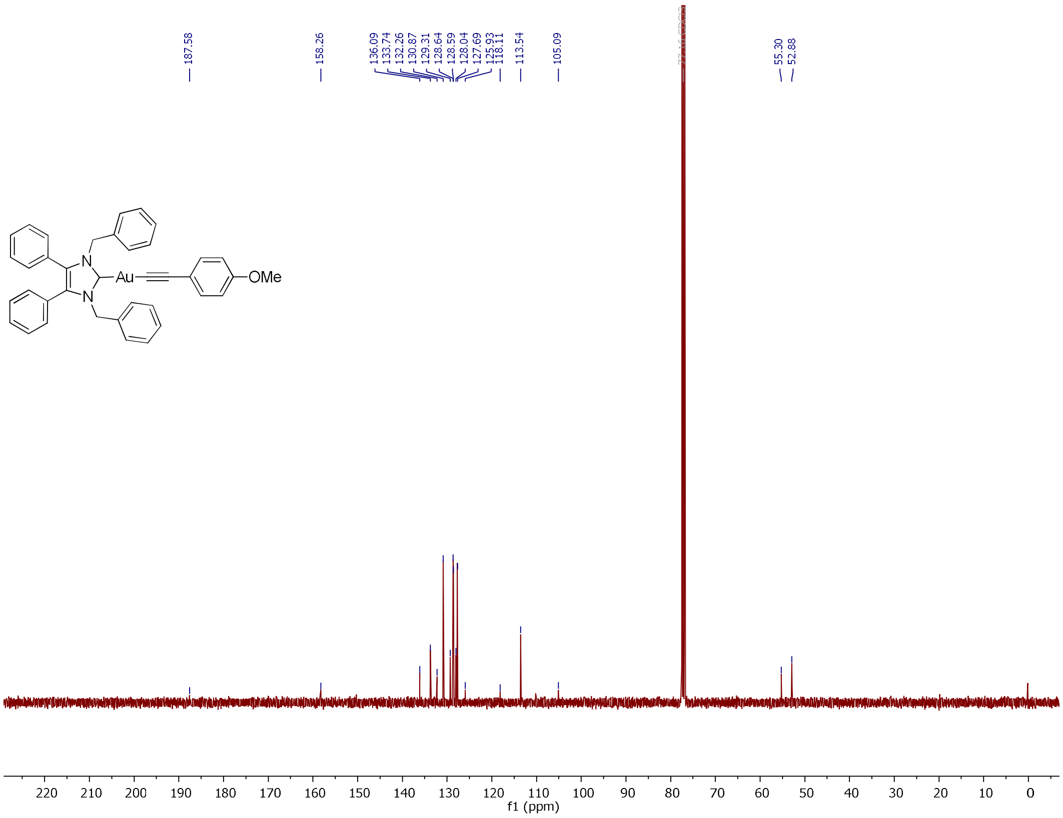

Figure S22. ^{13}C-NMR spectra of **5c** in $CDCl_3$.

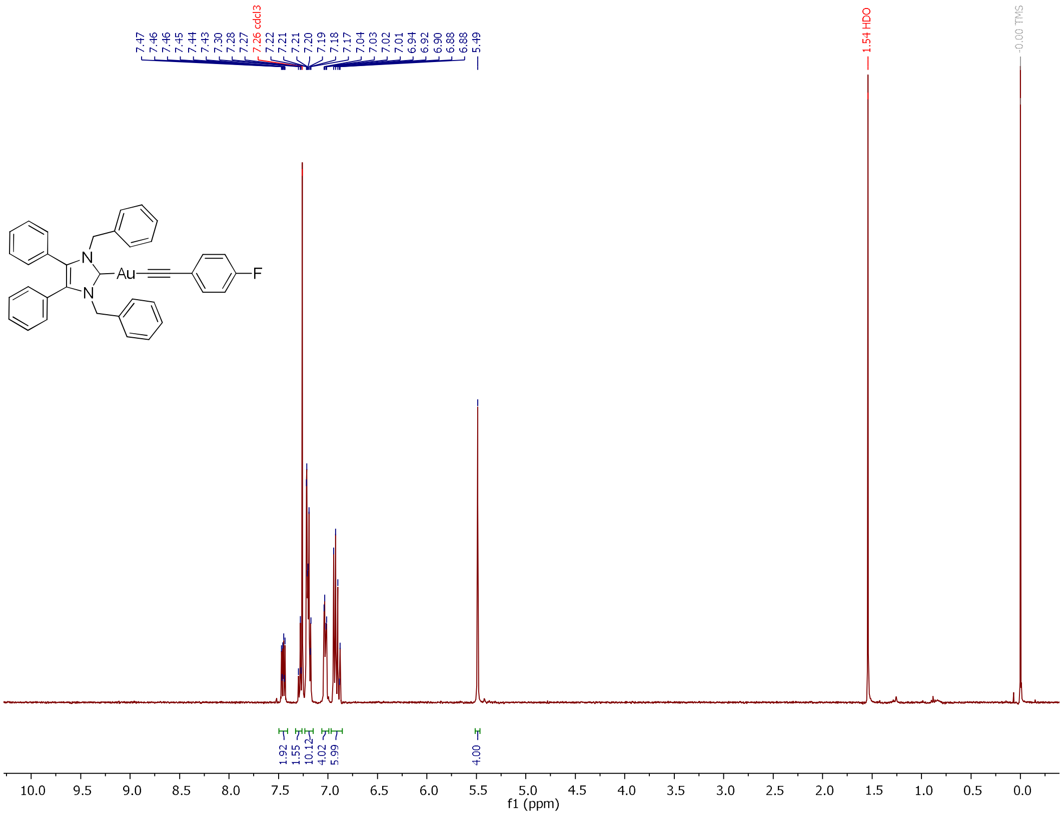

Figure S23. ^{1}H-NMR spectra of **5d** in $CDCl_3$.

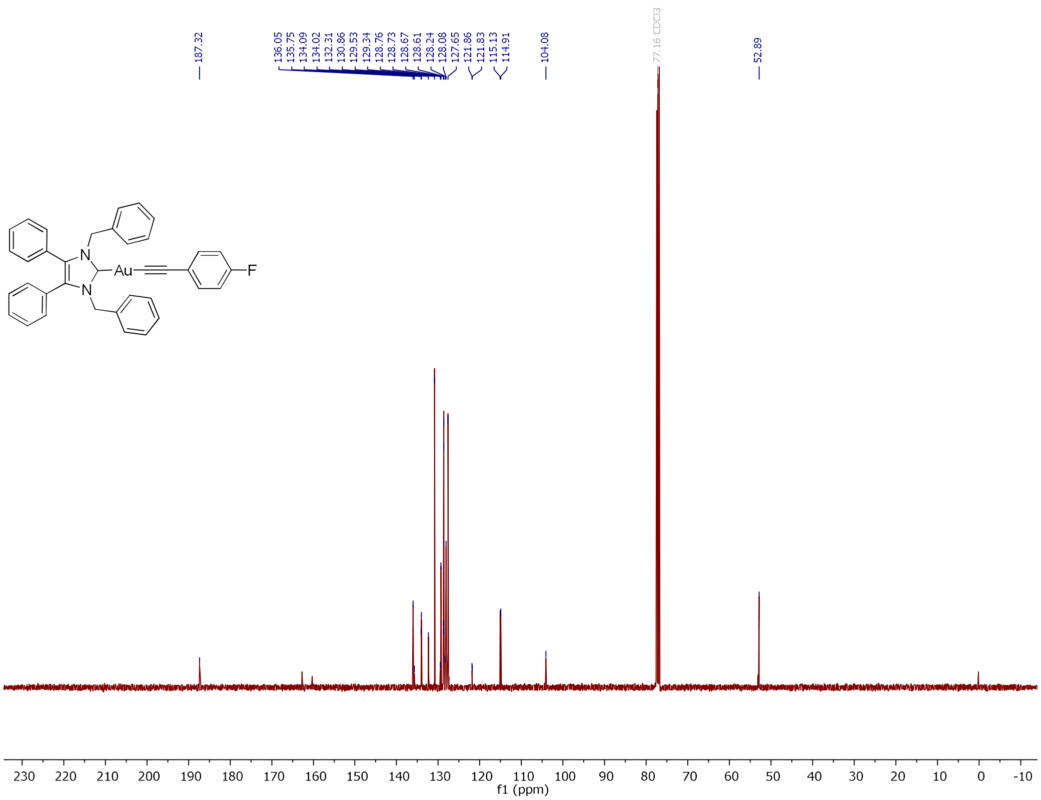

Figure S24. ^{13}C-NMR spectra of **5d** in $CDCl_3$.

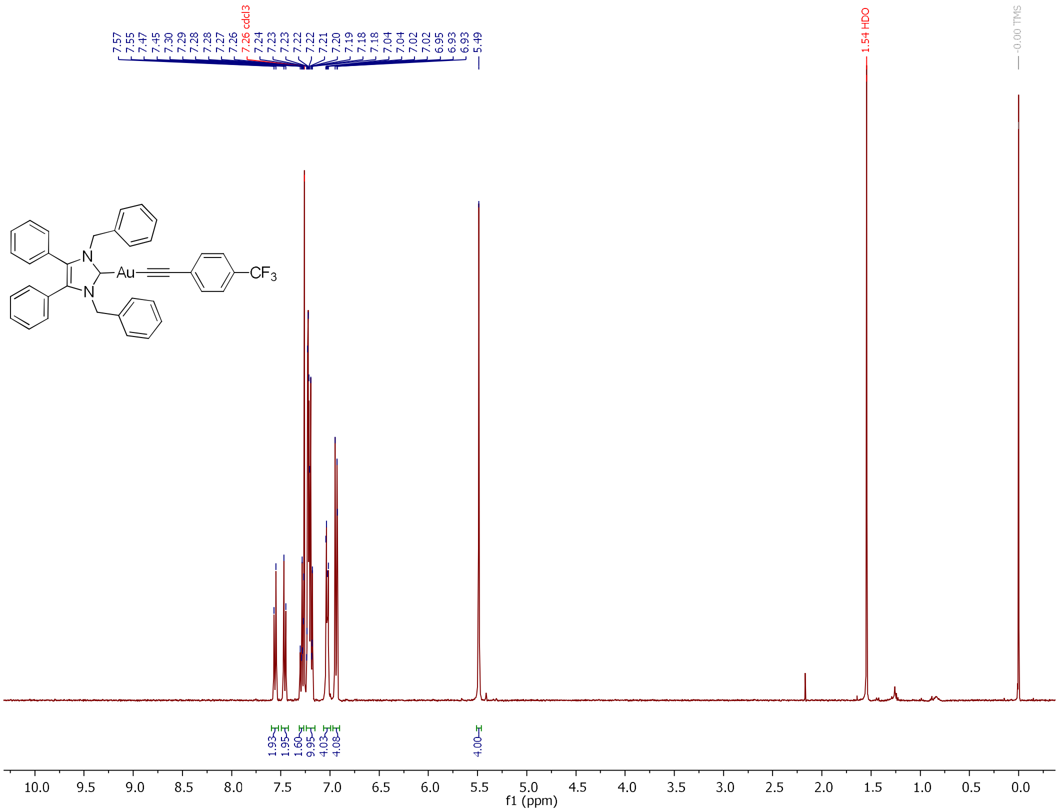

Figure S25. ^{1}H-NMR spectra of **5e** in $CDCl_3$.

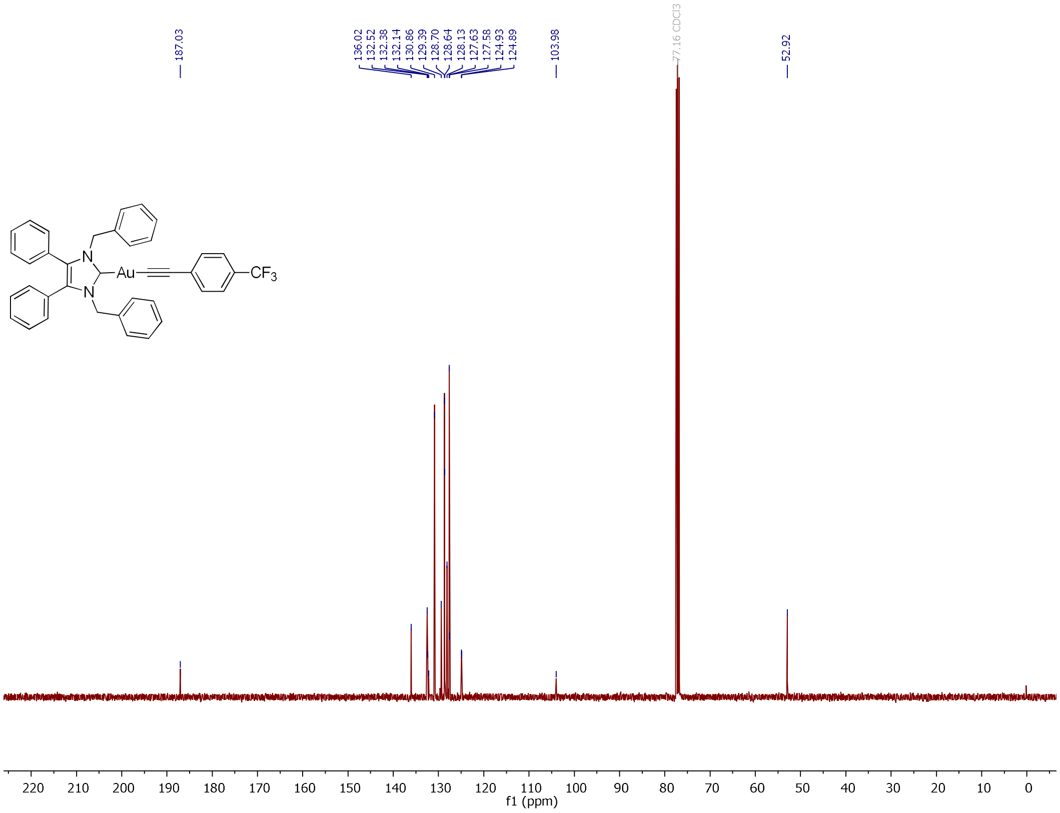

Figure S26. ^{13}C-NMR spectra of **5e** in $CDCl_3$.

Table S1. Crystal data and structure refinement for complexes **2b**-**3b**.

	**2b**	**2c**	**3a**	**3b**
Empirical Formula	$C_{30}H_{26}N_2Cl_2BrAu$	$C_{30}H_{26}N_2Cl_2IAu$	$C_{58}H_{48}N_4F_6PAu$	$C_{58}H_{48}BF_4N_4Au$
Formula Weight ($g\cdot mol^{-1}$)	762.30	809.29	1142.94	1084.78
Temperature (K)	100(2)	100(2)	100(2)	100(2)
Crystal system	Triclinic	Triclinic	Monoclinic	Triclinic
Space group	P–1 (#2)	P–1 (#2)	$P2_1/c$ (#14)	P–1 (#2)
Unit cell dimensions				
a (Å)	8.9019(2)	9.0569(2)	16.9459(1)	14.5025(1)
b (Å)	13.1121(3)	13.0587(2)	19.0314(1)	15.1574(1)
c (Å)	13.7953(3)	14.0021(3)	15.31484(8)	22.0115(2)
α (°)	112.593(2)	112.936(2)	90	90.4807(6)
β (°)	102.638(2)	102.247(2)	93.7614(5)	91.3160(6)
γ (°)	101.910(2)	102.555(2)	90	91.5461(5)
Volume ($Å^3$)	1373.18(6)	1405.14(5)	4928.46(5)	4835.34(6)
Z	2	2	4	4
Density (calcd) (mg/m^3)	1.844	1.913	1.540	1.490
Absorption coefficient (mm^{-1})	7.028	6.545	6.469	6.194
F (000)	736	772	2288	2176
Crystal size (mm^3)	0.263 x 0.151 x 0.093	0.256 x 0.161 x 0.062	0.261 x 0.061 x 0.043	0.129 x 0.101 x 0.078
θ (°)	2.956 to 30.508	2.989 to 32.858	3.496 to 77.108	3.527 to 76.829
Index ranges	$-12 \leq h \leq 12$ $-18 \leq k \leq 18$ $-19 \leq l \leq 19$	$-13 \leq h \leq 13$ $-19 \leq k \leq 19$ $-21 \leq l \leq 20$	$-21 \leq h \leq 21$ $-23 \leq k \leq 23$ $-14 \leq l \leq 19$	$-18 \leq h \leq 18$ $-18 \leq k \leq 19$ $-27 \leq l \leq 27$
Reflections collected	29326	44345	100377	131179
Independent reflections R_{int}	8375 (0.0421)	9743 (0.0278)	10384 (0.0504)	20239 (0.0405)
Completeness to θ_{max} (%)	99.8	99.8	100.0	100.0
Absorption correction	Gaussian	Gaussian	Gaussian	Gaussian
Max and min transmission	0.618 and 0.316	0.716 and 0.318	0.793 and 0.388	0.709 and 0.568
Refinement method	Full–matrix Least–squares on F^2	Full–matrix Least–squares on F^2	Full–matrix Least–squares on F2	Full–matrix Least–squares on F2
Data/ restraints/ parameters	8375 / 0 / 325	9743 / 0 / 325	10384 / 0 / 631	20239 / 0 / 1225
Goodness-of-fit on F2	1.040	1.063	1.037	1.051
Final R indices [$I > 2\sigma(I)$]	R1 = 0.0258 wR2 = 0.0524	R1 = 0.0189 wR2 = 0.0449	R1 = 0.0222 wR2 = 0.0546	R1 = 0.0254 wR2 = 0.0555
R indices (all data)	R1 = 0.0321 wR2 = 0.0550	R1 = 0.0203 wR2 = 0.0458	R1 = 0.0273 wR2 = 0.0581	R1 = 0.0322 wR2 = 0.0585
Largest diff. peak and hole	1.307 and –0.759	1.132 and –0.699	0.693 and –1.292	0.915 and –1.276

Table S2. Crystal data and structure refinement for complexes **4a**-**5b**.

	**4a**	**4b**	**5a**	**5b**
Empirical Formula	$C_{47} H_{39} N_2 F_6 P_2$ Au	$C_{47} H_{39} B N_2 F_4$ P Au	$C_{31} H_{25} N_2$ Au	$C_{37} H_{29} N_2$ Au
Formula Weight (g·mol⁻¹)	1004.71	946.55	622.49	698.59
Temperature (K)	100(2)	100(2) K	100(2)	100(2)
Crystal system	Triclinic	Triclinic	Monoclinic	Monoclinic
Space group	P–1 (#2)	P–1 (#2)	P21/c (#14)	$P2_1/n$ (#14)
Unit cell dimensions				
a (Å)	11.73747(8)	11.1534(2)	12.3303(3)	17.3520(3)
b (Å)	13.92645(7)	13.8188(2)	18.3303(3)	10.4155(1)
c (Å)	14.41601(7)	14.7889(2)	11.0923(2)	17.6623(2)
α (°)	80.3519(4)	81.470(1)	90	90
β (°)	66.5068(5)	68.978(2)	103.150(2)	114.610(2)
γ (°)	69.5446(5)	69.069(2)	90	90
Volume (Å^3)	2023.76(2)	1986.72(6)	2441.32(9)	2902.14(8)
Z	2	2	4	4
Density (calcd) (mg/m^3)	1.649	1.582	1.694	1.599
Absorption coefficient (mm^{-1})	8.128	3.798	6.048	9.730
F (000)	996	940	1216	1376
Crystal size (mm^3)	0.280 x 0.181 x 0.094	0.401 x 0.293 x 0.278	0.221 x 0.172 x 0.100	0.172 x 0.108 x 0.018
θ (°)	3.345 to 76.837	2.990 to 32.745	2.796 to 32.854	4.676 to 76.852
Index ranges	–12 ≤ h ≤ 14	–16 ≤ h ≤ 16	–18 ≤ h ≤ 17	–21 ≤ h ≤ 20
	–17 ≤ k ≤ 17	–20 ≤ k ≤ 20	–27 ≤ k ≤ 27	–13 ≤ k ≤ 12
	–18 ≤ l ≤ 18	–21 ≤ l ≤ 22	–16 ≤ l ≤ 16	–22 ≤ l ≤ 22
Reflections collected	78827	46327	76578	54931
Independent reflections R_{int}	8487 (0.0318)	13629 (0.0444)	8710 (0.0446)	6111 (0.0645)
Completeness to θ_{max} (%)	100.0	99.8	99.8	100.0
Absorption correction	Gaussian	Gaussian	Gaussian	Gaussian
Max and min transmission	0.539 and 0.245	0.457 and 0.380	0.601 and 0.377	0.845 and 0.363
Refinement method	Full–matrix Least–squares on F2	Full–matrix Least–squares on F2	Full–matrix Least–squares on F^2	Full–matrix Least–squares on F2
Data/ restraints/ parameters	8487 / 0 / 524	13629 / 0 / 505	8710 / 0 / 307	6111 / 0 / 361
Goodness-of-fit on F2	1.081	1.053	1.081	1.031
Final R indices [I > 2σ(I)]	R1 = 0.0192 wR2 = 0.0491	R1 = 0.0312 wR2 = 0.0683	R1 = 0.0246 wR2 = 0.0505	R1 = 0.0328 wR2 = 0.0836
R indices (all data)	R1 = 0.0197 wR2 = 0.0492	R1 = 0.0359 wR2 = 0.0709	R1 = 0.0331 wR2 = 0.0535	R1 = 0.0399 wR2 = 0.0903
Largest diff. peak and hole	1.202 and –0.714	3.089 and –0.932	1.321 and –0.610	1.857 and –1.785

Table S3. Crystal data and structure refinement for complexes **5c**-**5e**.

	**5c**	**5d**	**5e**
Empirical Formula	$C_{77}H_{64}N_4O_2Cl_2Au_2$	$C_{149}H_{114}N_8F_4Cl_2Au_4$	$C_{38}H_{28}F_3N_2Au$
Formula Weight (g·mol^{-1})	1542.15	2951.24	766.59
Temperature (K)	100(2)	100(2)	100(2)
Crystal system	Monoclinic	Monoclinic	Monoclinic
Space group	$P2_1/n$ (#14)	I2/a (#15)	P21/n (#14)
Unit cell dimensions			
a (Å)	15.0757(2)	31.4887(3)	14.9691(2)
b (Å)	9.14899(9)	9.06171(8)	10.91009(9)
c (Å)	23.2187(2)	42.0249(3)	19.2159(2)
α (°)	90	90	90
β (°)	93.0801(8)	98.2131(7)	90.0427(7)
γ (°)	90	90	90
Volume (Å^3)	3197.87(6)	11868.46(18)	3138.23(6)
Z	2	4	4
Density (calcd) (mg/m^3)	1.602	1.652	1.623
Absorption coefficient (mm^{-1})	9.665	10.007	9.199
F (000)	1524	5800	1504
Crystal size (mm^3)	0.121 x 0.066 x 0.020	0.201 x 0.047 x 0.027	0.151 x 0.108 x 0.039
θ (°)	3.413 to 76.907	3.292 to 76.873	3.742 to 76.879
Index ranges	$-18 \leq h \leq 18$	$-37 \leq h \leq 39$	$-18 \leq h \leq 18$
	$-11 \leq k \leq 11$	$-11 \leq k \leq 10$	$-13 \leq k \leq 13$
	$-29 \leq l \leq 29$	$-52 \leq l \leq 51$	$-24 \leq l \leq 24$
Reflections collected	34565	78037	63588
Independent reflections R_{int}	6711 (0.0383)	12388 (0.0507)	6602 (0.0516)
Completeness to θ_{max} (%)	100.0	99.9	100.0
Absorption correction	Gaussian	Gaussian	Gaussian
Max and min transmission	0.845 and 0.521	0.762 and 0.323	0.724 and 0.366
Refinement method	Full–matrix Least–squares on F^2	Full–matrix Least–squares on F^2	Full–matrix Least–squares on F2
Data/ restraints/ parameters	6711 / 0 / 407	12388 / 0 / 753	6602 / 0 / 397
Goodness-of-fit on F2	1.029	1.055	1.033
Final R indices [I > 2σ(I)]	R1 = 0.0209 wR2 = 0.0496	R1 = 0.0477 wR2 = 0.1204	R1 = 0.0361 wR2 = 0.0886
R indices (all data)	R1 = 0.0243 wR2 = 0.0515	R1 = 0.0540 wR2 = 0.1274	R1 = 0.0401 wR2 = 0.0915
Largest diff. peak and hole	0.770 and –0.851	3.002 and –2.638	3.496 and –1.281

Table S4. CCDC numbers for complexes **2b**-**5e**.

**Compound**	**Lab code**	**CCDC**
NHC*-Au-Br (**2b**)	Tac266	2012886
NHC*-Au-I (**2c**)	Tac262	2012885
[NHC*$_2$Au]PF_6 (**3a**)	Tac267	2012887
[NHC*$_2$Au]BF_4 (**3b**)	Tac271	2012888
[NHC*-Au-PPh_3]PF_6 (**4a**)	Tac279	2013114
[NHC*-Au-PPh_3]BF_4 (**4b**)	Tac287	2013115
NHC*-Au-CCH (**5a**)	Tac280	2012889
NHC*-Au-CC-Ph (**5b**)	Tac282	2012890

NHC*-Au-CC-Ph-*p*-OMe (**5c**)	Tac288	2012893
NHC*-Au-CC-Ph-*p*-F (**5d**)	Tac283	2012891
NHC*-Au-CC-Ph-*p*-CF_3 (**5e**)	Tac286	2012892

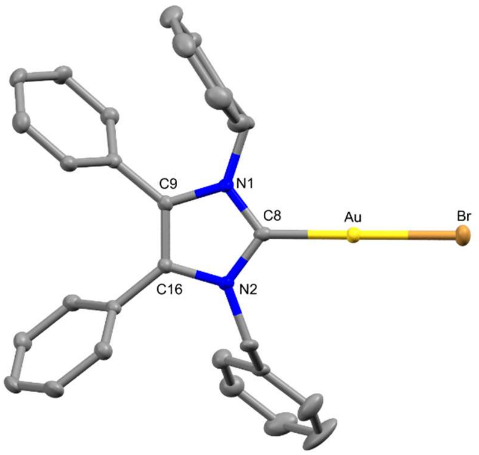

Figure S27. X-ray diffraction structures of NHC*-Au-Br (**2b)**; thermal ellipsoids drawn on the 50% probability level. Solvent molecules and hydrogen atoms have been omitted for clarity.

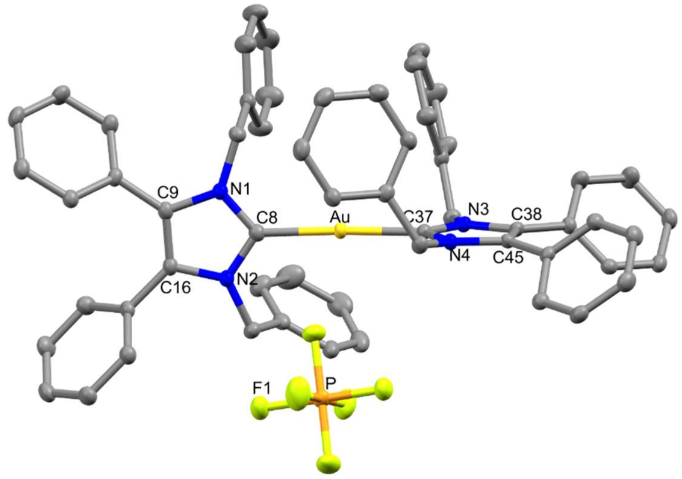

Figure S28. X-ray diffraction structures of [NHC*$_2$Au]PF_6 (**3a**); thermal ellipsoids drawn on the 50% probability level. Hydrogen atoms have been omitted for clarity.

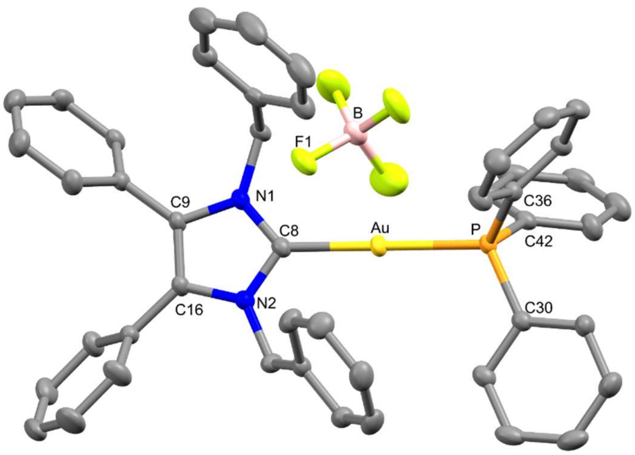

Figure S29. X-ray diffraction structures of [NHC*-Au-PPh_3]BF_4 (**4b**); thermal ellipsoids drawn on the 50% probability level. Hydrogen atoms have been omitted for clarity.

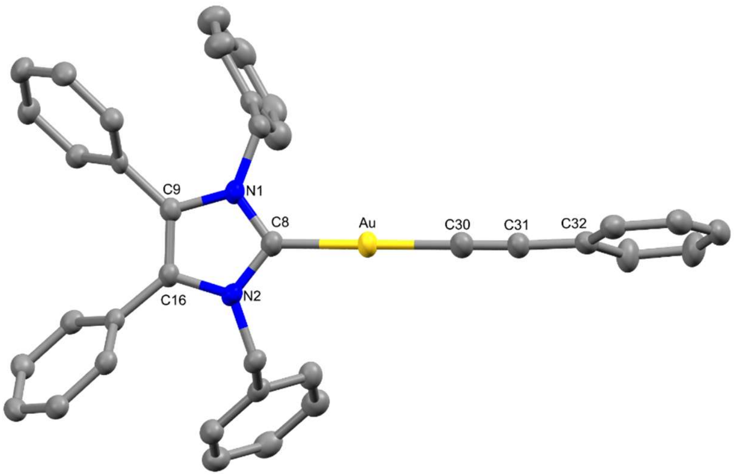

Figure S30. X-ray diffraction structures of NHC*-Au-CC-Ph (**5b**); thermal ellipsoids drawn on the 50% probability level. Hydrogen atoms have been omitted for clarity.

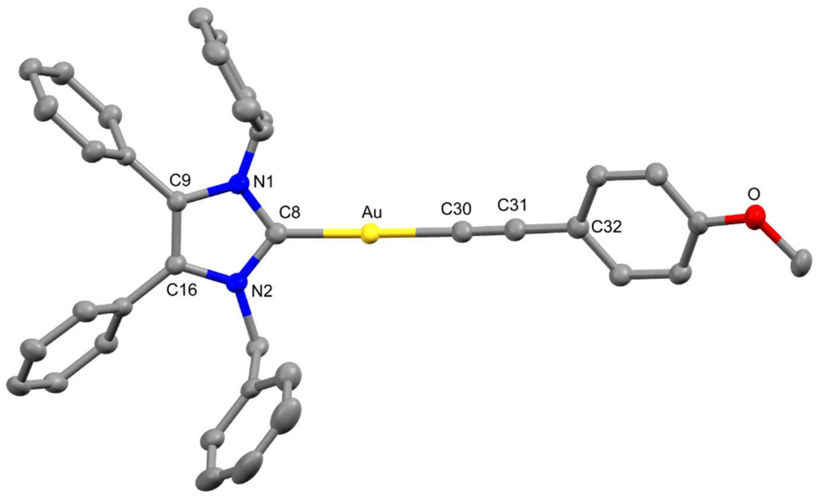

Figure S31. X-ray diffraction structures of NHC*-Au-CC-Ph-*p*-OMe (**5c**); thermal ellipsoids drawn on the 50% probability level. Solvent molecules and hydrogen atoms have been omitted for clarity.

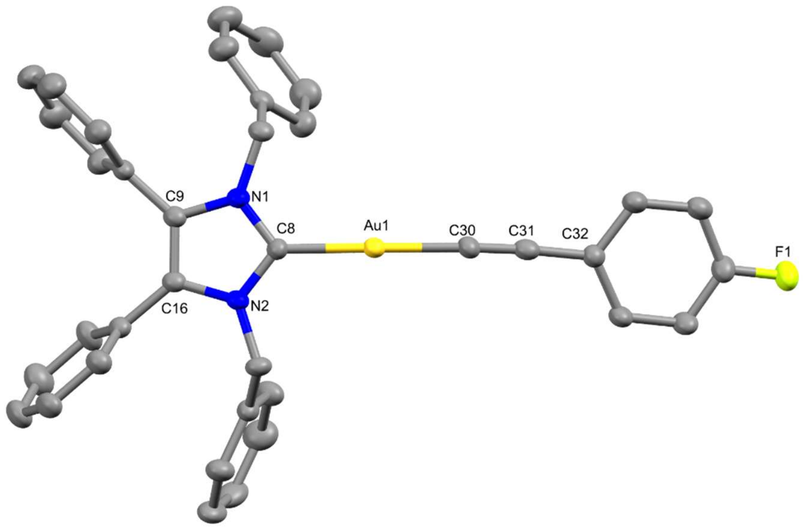

Figure S32. X-ray diffraction structures of NHC*-Au-CC-Ph-*p*-F (**5d**); thermal ellipsoids drawn on the 50% probability level. Solvent molecules and hydrogen atoms have been omitted for clarity.

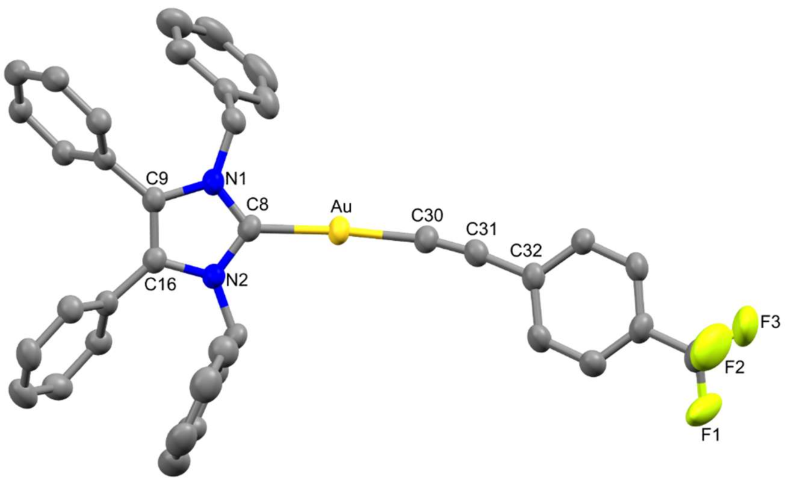

Figure S33. X-ray diffraction structures of NHC*-Au-CC-Ph-*p*-CF_3 (**5e**); thermal ellipsoids drawn on the 50% probability level. Hydrogen atoms have been omitted for clarity.
